# Bioengineering Outer-Membrane Vesicles for Vaccine Development: Strategies, Advances, and Perspectives

**DOI:** 10.3390/vaccines13070767

**Published:** 2025-07-20

**Authors:** Ayesha Zahid, Hazrat Ismail, Jennifer C. Wilson, I. Darren Grice

**Affiliations:** 1 Institute for Biomedicine and Glycomics, Griffith University, Gold Coast, QLD 4222, Australia; 2 School of Pharmacy and Medical Science, Griffith University, Gold Coast, QLD 4222, Australia; jennifer.wilson@griffith.edu.au; 3 MOE Key Laboratory for Membraneless Organelles & Cellular Dynamics and CAS Center for Excellence in Molecular Cell Science, Hefei National Laboratory for Physical Sciences at the Microscale, University of Science and Technology of China, Hefei 230027, China; ismail@mail.ustc.edu.cn

**Keywords:** outer membrane vesicles, bioengineered vaccines, vaccine development, genetic engineering, cancer vaccines, multi-antigen vaccines, multi-pathogen vaccines

## Abstract

Outer-membrane vesicles (OMVs), naturally secreted by Gram-negative bacteria, have gained recognition as a versatile platform for the development of next-generation vaccines. OMVs are essential contributors to bacterial pathogenesis, horizontal gene transfer, cellular communication, the maintenance of bacterial fitness, and quorum sensing. Their intrinsic immunogenicity, adjuvant properties, and scalability establish OMVs as potent tools for combating infectious diseases and cancer. Recent advancements in genetic engineering and biotechnology have further expanded the utility of OMVs, enabling the incorporation of multiple epitopes and antigens from diverse pathogens. These developments address critical challenges such as antigenic variability and co-infections, offering broader immune coverage and cost-effective solutions. This review explores the unique structural and immunological properties of OMVs, emphasizing their capacity to elicit robust immune responses. It critically examines established and emerging engineering strategies, including the genetic engineering of surface-displayed antigens, surface conjugation, glycoengineering, nanoparticle-based OMV engineering, hybrid OMVs, and in situ OMV production, among others. Furthermore, recent advancements in preclinical research on OMV-based vaccines, including synthetic OMVs, OMV-based nanorobots, and nanodiscs, as well as emerging isolation and purification methods, are discussed. Lastly, future directions are proposed, highlighting the potential integration of synthetic biology techniques to accelerate research on OMV engineering.

## 1. Introduction

Living organisms naturally secrete biomolecules such as polysaccharides, proteins, and various chemical compounds, either directly or through indirect pathways, to support their physiological functions [1]. In addition to the direct release of certain cellular components, numerous factors are released in the form of vesicles, suggesting that these vesicles could significantly influence various biological functions. Outer-membrane vesicles (OMVs) spontaneously bud from the cell envelope of Gram-negative bacteria [2]. OMV generation was initially documented in 1965 in an auxotrophic strain of *Escherichia coli* that emitted substantial quantities of cell-free LPS under lysine-deficient growth settings [3]. Subsequently, Knox and colleagues reported that the released cell-free LPS components were integral to membrane structures, and they hypothesized that these vesicles originate from the outer membrane [4]. Rothfield and Pearlman-Kothencz further showed that chloramphenicol exposure and amino acid deprivation enhanced outer membrane blebbing in *E. coli* [5]. Following that, studies documented the observation and separation of OMVs from various Gram-negative bacteria, including *Veillonella parvula* [6], *Vibrio cholerae* [7], and *Salmonella enterica* ser. Typhimurium [5]. Notwithstanding the mounting evidence of OMV generation by bacteria, OMVs were regarded as trivial artefacts of growth or by-products of cell lysis for many years. Finally, OMVs were detected in cerebral fluid samples from patients with acute meningitis, indicating that OMVs are not exclusively produced in laboratory settings [8]. Since then, the synthesis of OMVs and their functions have garnered significant interest.

**Figure 1:**
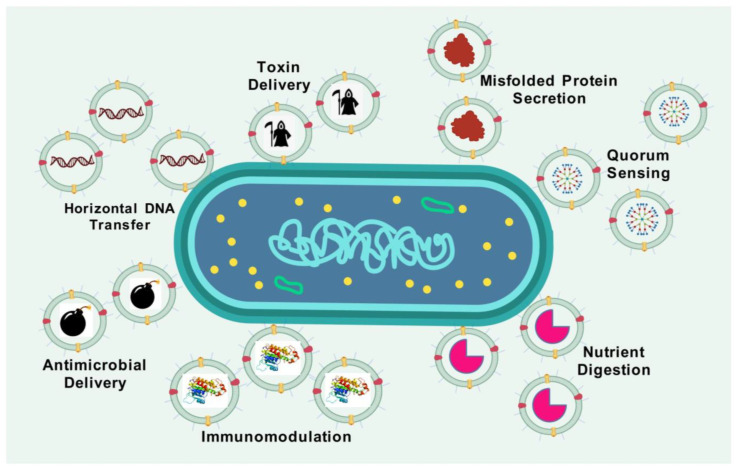
OMVs: Small packages with large roles in bacterial physiology. OMVs originate from the outer membrane of parent bacteria and inherently encapsulate numerous cellular substances. The bio-functions of OMVs are classified and delineated as follows: toxin secretion, horizontal DNA transfer, antimicrobial delivery, immunomodulation, nutrition digestion, quorum sensing, and secretion of misfolded proteins.

OMVs are inherently generated by all Gram-negative bacteria throughout their usual growth cycle. The identification of OMVs across diverse growth parameters and natural settings in all examined bacteria suggests that OMV biogenesis is an evolutionarily conserved mechanism [9]. OMVs are secreted by both pathogenic and non-pathogenic bacteria. While they may contribute to disease pathogenesis, their non-replicative nature prevents them from independently inducing disease. Their formation and composition are influenced by temperature, growth conditions, bacterial growth stage, and quorum sensing [10]. The quantity of OMV production and the elements transported by OMVs vary, even among genetically identical bacteria across different environments [11]. Comprehending the biogenesis and biological roles of OMVs is critically important, as it provides new avenues for their application. Proteomic analyses and electron microscopy have established that OMVs are diverse nanostructures encapsulating a variety of bioactive substances [12,13,14]. OMVs function as intermediaries, facilitating the transfer of biological information from bacteria to the host. They deliver short RNAs to enhance host–pathogen interactions [15]. Moreover, OMVs may function as a conduit for antibiotic resistance genes [16] or virulence factors [17]. For example, OMVs from *Bacteroides thetaiotaomicron* transport cephalosporinases to shield gut pathogens from β-lactam antibiotics [18]. Furthermore, OMVs can assimilate into biofilms, facilitating biofilm development by supplying essential nutrients [19]. OMVs also function as a versatile secretion mechanism for bacteria [20]. One hypothesis suggests that bacteria generate OMVs as a defence mechanism in response to external dangers, such as bacteriophage infection [21], antimicrobial peptides [22], and antibiotics [18], underscoring the significant role of OMVs in bacterial pathogenesis. For example, *Porphyromonas gingivalis* OMVs impair host immune responses by selectively inducing tolerance to tumour necrosis factor (TNF-α) production in monocytes via a Toll-like receptor 4 (TLR4)- and mTOR-dependent mechanism, thereby promoting immune evasion and persistence [23]. OMVs may sometimes be produced due to physiological imbalances, such as a mismatch between cell proliferation and outer membrane synthesis, resulting in excess membrane material that is shed as OMVs. A summary of the various functions of OMVs is illustrated in Figure 1.

Due to their exceptional biocompatibility, enhanced stability, and adaptability for modification, OMVs have become key components in vaccine development. Given the rapid advancements in this field, providing an updated perspective on their potential vaccine applications is essential. In this review, the structure and composition of OMVs are explored, followed by a discussion of current strategies and recent advancements in their modification for vaccine applications. Additionally, emerging trends in preclinical studies and future perspectives in the field are highlighted. In doing so, this review provides valuable insights that may guide the development of more effective and innovative OMV-based vaccines.

## 2. The Formation, Structure, and Composition of OMVs

**Figure 2:**
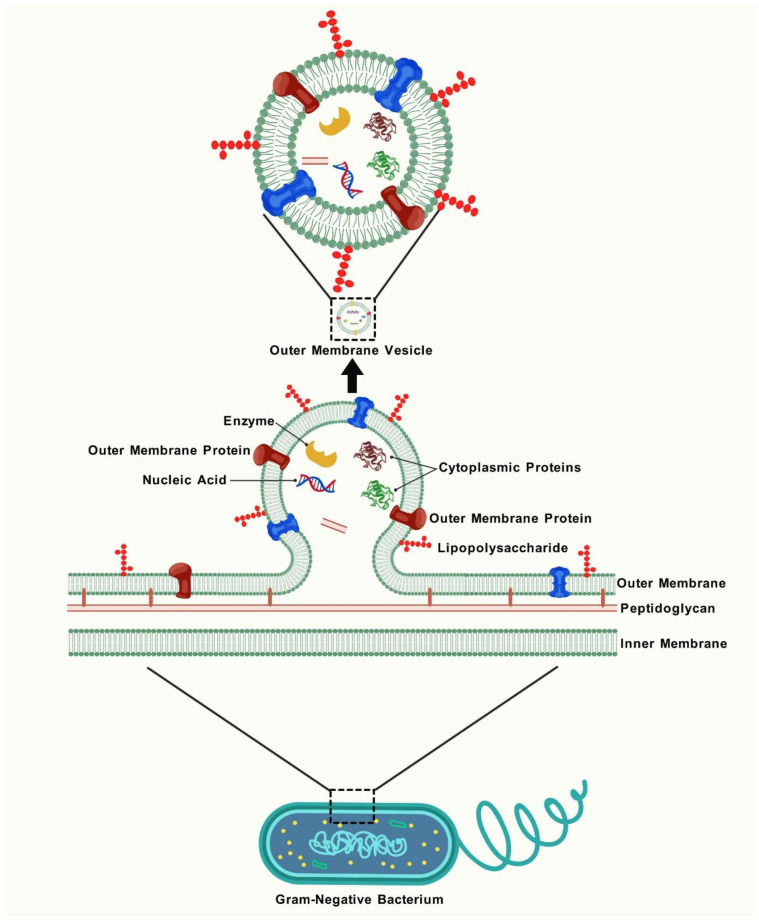
Biogenesis and composition of OMVs. An OMV comprises an inner membrane and an outer membrane. Phospholipids, lipopolysaccharides, and outer-membrane proteins are present in the outer membrane. Phospholipids and integral membrane proteins are prevalent in the inner membrane. OMVs carry internal constituents such as DNA and RNA, along with outer-membrane proteins and components from the periplasmic space.

Despite decades of investigation, scientists have been unable to clarify a clear or universal process for OMV formation. At least five ways have been suggested to elucidate the formation of OMVs. The main events involved are as follows: (a) disruption of the connections between the peptidoglycan layer and the outer membrane; (b) buildup of substances in the periplasmic space; (c) activation of certain signalling and activator molecules [24]; and then (d) the impacted areas of the outer membrane subsequently extend to create vesicular blebs, which persistently extend outward until they separate from the residual OM, resulting in the formation of OMVs [1], as shown in Figure 2. The biogenesis of OMVs has been reviewed in detail elsewhere [25,26]. As extensions of the outer membrane of the parent bacterium, OMVs acquire a similar composition [27], encompassing the surface intricacy in a manner unattainable by vaccines derived from recombinant protein antigens. The structure and constituents of OMVs, as illustrated in Figure 2, underpin their functionality and render them immunostimulatory and accessible to immune cells, imparting inherent immunogenicity and adjuvanticity to OMVs. The usual size of OMVs is 20–250 nm; however, in some instances, the diameter range expands to 500 nm and may encompass diverse irregular shapes, analogous to the diversity seen in numerous enveloped viruses [28]. The membrane of OMV comprises phospholipids (PL) internally and lipopolysaccharides (LPS) and PL externally, interspersed with membrane proteins in diverse orientations, predominantly mirroring the architecture of the outer membrane [29]. LPS, a significant pathogen-associated molecular pattern (PAMP), is present in high amounts in OMVs. Typically, the primary type of LPS found in OMVs is B-band LPS, which is linked to a charged polysaccharide and plays a key role in OMV formation [30]. PLs mostly consist of phosphatidyl ethanolamine and phosphatidyl glycerol, although they exhibit variability in OMVs among distinct bacterial species. Phosphatidyl ethanolamine is the predominant phospholipid in the OMVs of *E. coli*, whereas it is found in comparatively low concentrations in the OMVs of *Helicobacter pylori*, where diphosphatidyl glycerol serves as the principal phospholipid [31,32].

The lumen of the vesicle may encompass many molecules from the periplasm or cytoplasm, including proteins, RNA/DNA, and peptidoglycan [12], as shown in Figure 2. The mechanism by which cytoplasmic components, including DNA and RNA, are carried into the periplasm for incorporation into OMVs remains ambiguous [33,34]. The analysis of outer-membrane and OMV fractions from various bacteria demonstrates a specific set of proteins and lipids in each fraction [35,36,37]. These findings suggest that bacteria have distinct sorting processes for OMVs and that OMV production is a planned activity rather than a result of cell lysis.

In proteomic analyses of OMVs, the total number of detected proteins ranged from 50 to 338, contingent upon the methodology and bacterial species, with 40–80% anticipated to represent outer membrane proteins [32,38,39,40,41]. The proteins found in OMVs can be classified into three categories: (i) fundamental proteins of the outer membrane, (ii) specialized cargo proteins within the lumen, and (iii) unidentified or contaminating proteins. The first category includes major outer membrane proteins, such as porins, adhesins, components of transport systems, enzymes like phospholipases and proteases, and pilus or flagellum proteins. Adhesins facilitate the attachment of OMVs to designated target cells [42,43]. The second group of specialized cargo proteins may encompass various toxins, for example, cholera toxin from *V. cholerae* [44], cytolysin A from enterotoxic *E. coli* [45,46], cytolethal distending toxin (CDT) from *Campylobacter jejuni* [47], and cholera toxin vacuolating cytotoxin (VacA) from *H. pylori* [48], and enzymes including ureases and proteases. The third category of proteins is identified as comprising components from other cellular regions that are not regarded as standard OMV constituents; however, the presence of these proteins is expected to diminish with improved isolation techniques [49,50]. In addition, OMVs also comprise small RNA (sRNA) and transfer RNA (tRNA) fragments (~35 nt) crucial for regulating host gene expression through binding to mRNA transcripts, hence influencing transcription and translation stability [51]. Choi et al. demonstrated that sRNAs present in OMVs from *Aggregatibacter actinomycetemcomitans*, *Treponema denticola*, and *P. gingivalis* reduce cytokine secretion by Jurkat T-cells, hence inhibiting immunological response [15]. Details of OMV sRNA–host interactions have been covered in detail elsewhere [52]. Identifying and characterizing novel components within OMVs that specifically influence human immune responses to infection could lead to innovative treatment strategies for combating chronic bacterial infections and preventing the emergence of antibiotic resistance.

OMVs are heterogeneous in size, protein composition, and content, shaped by both endogenous and exogenous factors. Endogenous factors include bacterial strain and biogenesis route, while exogenous factors involve growth conditions, extraction method, and growth stage. These variations can influence the OMV targeting of specific host cells and their resulting biological effects. For instance, a recent study showed that the bacterial growth phase affects OMV-induced IL-8 production in human epithelial cells [53]. OMVs generated from *V. cholerae* exhibit heterogeneity in size, influenced by varying culture environments and purification techniques that also impact the chemical content of the OMVs [54]. *Stenotrophomonas maltophilia* stimulated with ciprofloxacin produced more vesicles, which were larger in size and contained more cytosolic proteins, compared to the non-stimulated cells [55]. The absence of uniformity in the production parameters and extraction methods can result in significant variability in the composition and size of OMVs among different batches, hence impacting their clinical applicability. Therefore, transmission electron microscopy, nanoparticle tracking analysis, and mass spectrometry are frequently employed to examine the morphology, ascertain the particle size and zeta potential, and assess the composition of OMVs, respectively [56]. Although a universal set of OMV-specific proteins has not yet been defined, proteomic analyses have shown the enrichment of outer-membrane and periplasmic proteins that may offer potential as characteristic markers [57]. Therefore, a supplementary quality control measure for future OMV-based biomaterials may involve the identification of distinctive and consistently present proteins within OMVs, which could serve as a “protein fingerprint” for batch verification and product consistency.

## 3. Vaccine Potential of OMVs

### 3.1. Distinctive Features of OMVs for Vaccine Development

Vaccines stimulate a strong and enduring pathogen-specific immune response by imitating a pathogen without inducing the resulting disease, therefore activating both innate and adaptive immunity. A vaccine must contain three essential components: (i) pathogen-specific antigens, (ii) numerous PAMPs, and (iii) an appropriate size to stimulate the immune system [58].

**Figure 3:**
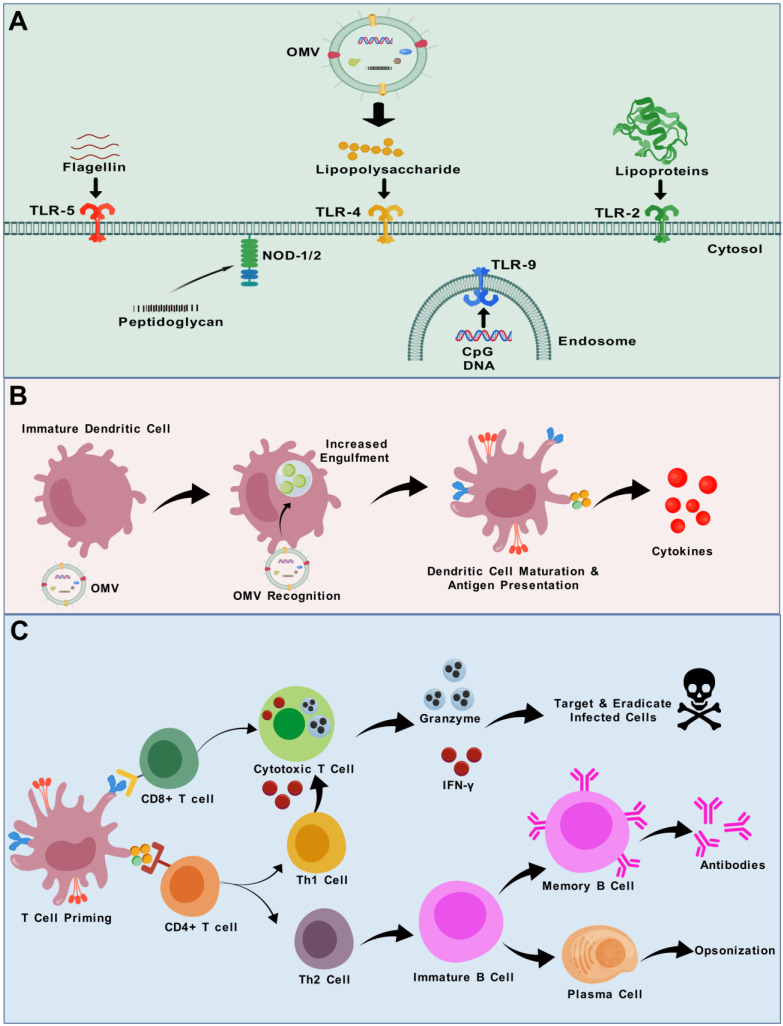
Harnessing OMVs for vaccination: bridging innate and adaptive immunity. (**A**) Identification of OMV-related pathogen-associated molecular patterns (PAMPs) by pattern recognition receptors (PRRs). Arrows denote ligand–receptor interactions between PAMPs and NOD-like receptors (NLRs) or Toll-like receptors (TLRs). (**B**) Upon delivery, OMV vaccines, including antigens and diverse PAMPs, are identified and engulfed by immature dendritic cells after the interaction of PAMPs with PRRs. The identification and encapsulation of OMV vaccines by dendritic cells promote their development, characterized by the production of co-stimulatory molecules and cytokine release, as well as antigen presentation. (**C**) Mature dendritic cells presenting bacterial antigens stimulate the activation and proliferation of antigen-specific CD4^+^ and CD8^+^ T lymphocytes in lymph nodes. In response to distinct cytokine environments, CD4^+^ T-cells differentiate into Th1 and Th2 cells. Th2 cells assist B-cells in generating antigen-specific antibodies that adhere to, and facilitate the elimination of, germs through opsonization. CD8^+^ cytotoxic T lymphocytes (CTLs) identify and eliminate bacteria-infected cells by exerting cytotoxic effects and releasing cytokines, including IFN-γ, upon encountering bacterial antigens. The efficacy of CTLs is augmented by Th1 cells via the secretion of IFN-γ. B-cell: B lymphocyte (Bursa-derived cell); CpG: Cytosine-phosphate-Guanine (CpG) dinucleotides; CD4^+^ T-cells: Cluster of differentiation four positive T-cells; CD8^+^ T-cells: Cluster of differentiation eight positive T-cells; IFN-γ: Interferon gamma; NOD-1/2: NOD-like receptor 1/2; TLR-2: Toll-like receptor 2; TLR-4: Toll-like receptor 4; TLR-5: Toll-like receptor 5; TLR-9: Toll-like receptor 9; T-cell: T lymphocyte (Thymus-derived cell); Th1 cell: T helper 1 cell; Th2 cell: T helper 2 cell; OMV: outer-membrane vesicle.

OMVs serve as a bridge between conventional and modern vaccine production techniques, offering a practical solution for combating a range of diseases [59]. Remarkably, OMVs concurrently fulfil all three of the essential criteria for an effective vaccine outlined above. OMVs possess a library of antigens in their natural form, which could offer enhanced and more comprehensive protection. They inherently possess various PAMPs and pathogen-associated antigens. Due to their small size, they can access lymph nodes via lymphatic drainage or through phagocytosis, subsequently being transported by antigen-presenting cells (APCs) (Figure 3) [60]. They are non-replicative particles; therefore, they cannot induce disease. Furthermore, OMVs possess inherent adjuvant characteristics that significantly enhance innate and adaptive immune responses [60]. The remarkable stability of OMVs under elevated temperatures and various chemical treatments underscores their potential as promising vaccine candidates [61]. Their small size and particle morphology promote systemic distribution and evoke long-term memory responses [62]. Conventional inactivated vaccines and subunit vaccines can contain denatured proteins and other constituents as a result of the manufacturing process, therefore reducing the antigenic diversity of the vaccine. In contrast, OMV-based vaccines provide novel solutions by displaying native, multivalent antigens in their inherent conformations, hence augmenting their immunogenicity. Importantly, they can incorporate multiple conserved epitopes from the target pathogen, addressing antigenic variability and reducing the likelihood of immune evasion [63]. The manufacturing process of OMVs is relatively simple, relying on the cultivation of bacteria and the filtration of OMVs released in the culture supernatant. Consequently, OMV technology appears especially appealing for developing cost-effective vaccines, a crucial factor for immunization programmes, particularly in low- and middle-income regions [64]. Finally, bioengineering allows for the incorporation of tailored antigens in OMVs, thereby expanding their potential against various diseases. These benefits establish OMVs as a viable platform to address the shortcomings of conventional vaccine methods, especially for diseases exhibiting intricate immune evasion mechanisms or significant antigenic variation. A comparison of the strengths and limitations of the OMV-based vaccine platform with other vaccine platforms is provided in Table 1.

### 3.2. Adjuvant Activity of OMVs

OMVs function as adjuvants due to the abundant presence of PAMPs, stimulation of danger signals, and unique geographical framework. PAMPs present in OMVs activate both Toll-like receptors (TLRs) and pattern recognition receptors (PRRs), which eventually induce APCs. Therefore, OMVs may enhance the antigen absorption and surface expression of immunostimulatory elements, facilitating T-cell development [77], as shown in Figure 3. PAMPs present in OMVs influence host cells, resulting in an augmented immune reaction and maturation of T-cells [78]. The geographic framework refers to the transport of antigenic components to the tissue-draining lymph node from the site of injection through dendritic cells. Hence, OMVs possess immunogenic characteristics, function as carriers, and exhibit natural adjuvant activities [79]. Numerous infectious pathogens impact mucosal tissue; hence, vaccines aimed at mucosal tissue are highly advantageous. Conventional adjuvants are insufficient for delivering antigens to the mucosal area, while OMV-based vaccines are showing promise in this aspect. Using a lipid raft-dependent endocytic route by epithelial cells, OMVs are internalized and subsequently sorted in lysosomes [80]. Therefore, they operate as mucosal transporters that convey antigens activating APCs. Among various immune effects, the LPS component of OMVs can activate B lymphocytes via TLR4, and, in some cases, through dual-signalling with the B-cell receptor (BCR), promoting antibody responses [81]. OMVs can stimulate B-cells through additional pathways as well [82]. Hence, mucosal OMV-based adjuvants act as a transporter of antigens and elicit an immunological response [83]. Integrating OMV adjuvants into novel or established vaccines has the potential to significantly enhance the scale and scope of adaptive immune responses, hence improving vaccine effectiveness [84]. The adjuvant efficacy of OMVs has been examined through their combination with model antigens. For example, compared to keyhole limpet hemocyanin (KLH) alone, the combination of KLH with OMVs significantly increased both KLH-specific antibody and CD4^+^ T-cell responses, which was attributed to the ability of OMVs to activate dendritic cells via TLR4 signalling, upregulating the co-stimulatory molecules and proinflammatory cytokines that promote effective antigen presentation [85]. OMVs have also demonstrated adjuvant activity for the hepatitis B virus surface antigen (HBsAg), leading to a vigorous induction of HBsAg-specific IgA and IgG responses [86]. These findings effectively highlight the adjuvant potential of OMVs.

### 3.3. Therapeutic Potential of OMVs for Cancer/Tumour Vaccines

In the extensive pursuit of combating cancer, researchers have also focused on cancer prevention and prognostic management, including the development of tumour vaccines. A tumour vaccine, also referred to as a cancer vaccine or therapeutic cancer vaccine, is a form of immunotherapy intended to activate the immune system to identify and target cancer cells. In comparison to traditional therapies, cancer vaccines provide increased reliability and effectiveness [87]. Nonetheless, contemporary cancer vaccines exhibit some notable drawbacks, including limited immunogenicity, complex production processes, challenges and high costs associated with development, and, most critically, an increasing requirement for vaccine carriers [87]. In recent decades, OMVs have become a fundamental component in novel strategies for cancer treatment. In contrast to live or attenuated bacteria, OMVs are considered a safe alternative for cancer vaccines due to their inability to self-replicate within the host organism. OMVs exhibit remarkable resistance to temperature fluctuations and contain numerous immunogenic components. These components possess the capacity to provoke a widespread immunological response in host cells. These intrinsic characteristics render OMVs exceptionally qualified for applications ranging from vaccines to immune modulators in innovative cancer therapy methodologies [27].

The prevailing perspective suggests that tumour cells, due to their increased demand for nutrients and oxygen to support accelerated proliferation, secrete vascular endothelial growth factors (VEGFs) and other growth factors involved in tumour angiogenesis. The newly formed tumour blood vessels exhibit notable morphological and structural differences compared to normal vessels, including large gaps between endothelial cells, the absence of a smooth muscle layer in the vessel wall, and impaired angiotensin receptor function. Moreover, tumour tissues lack lymphatic vessels, leading to impaired lymphatic fluid drainage [88]. These structural alterations facilitate the passage of large molecules across the arterial walls, allowing for them to accumulate at tumour sites for extended periods by evading lymphatic clearance. This phenomenon, known as the enhanced permeability and retention (EPR) effect in solid tumours, enables the passive targeting of OMVs [89]. Kinetic investigations indicate that OMVs infiltrate blood vessels through systemic circulation post-administration, navigate the tumour vasculature, and persist at the tumour site due to the EPR effect, highlighting the potential of drug-laden OMVs for targeted tumour therapy [90]. The hypoxic microenvironment is a prevalent characteristic in around 90% of solid tumours [91]. It is intricately linked to tumour proliferation, differentiation, angiogenesis, and energy utilization, as well as the development of resistance to therapy and adverse patient prognosis, serving as a primary factor in the difficult elimination of cancer cells [92]. Some live bacteria inherently display tumour tropism, owing to their affinity for the hypoxic environment [93]. Consequently, OMVs released by such bacteria may selectively identify intratumoural germs via a homing effect, be biocompatible, and cause no harm to surrounding healthy tissues. Consequently, OMVs released by such bacteria may selectively identify intra-tumoural germs via a homing effect, be biocompatible, and cause no harm to surrounding healthy tissues. For example, *E. coli*-derived OMVs displaying an anti-HER2 affibody on their surface via ClyA fusion have shown selective targeting of HER2-positive cancer cells and successful delivery of therapeutic siRNA [94]. Overall, OMVs have encouraging natural tropism for malignant tumours, utilizing both aggressive and passive targeting mechanisms [95].

Antitumour vaccines utilizing OMVs predominantly depend on sophisticated genetic engineering methodologies. These strategies involve the integration of antigens within the vesicle lumen or on its surface, eliciting the desired immune reaction while not compromising the initial immunogenicity or causing adverse effects. Fortunately, the latest advancement in OMV engineering can allow for the presentation of various tumour antigens on the surface of OMVs. Therefore, it may be possible to develop a flexible OMV-based platform that can swiftly and concurrently display many antigens for the tailored development of tumour vaccines.

### 3.4. OMVs and Trained Immunity

Pre-inoculation with OMVs can augment tumour vaccination through a mechanism referred to as trained immunity. The induction of trained immunity enhances the innate immune system’s potency for a specific duration, resulting in an amplified response to future immunological stimuli. This phenomenon is facilitated by immune cells like natural killer cells or macrophages which can be reprogrammed by specific stimuli to provide either a more or less vigorous and effective immune response [96]. The memory-like ability of innate immune cells can be utilized to enhance the effectiveness of future vaccinations. A recent study demonstrated that OMVs derived from *E. coli* BL21 can enhance the efficacy of tumour vaccines through the induction of trained immunity. In this model, mice pretreated with OMVs one week before receiving a tumour vaccine showed significantly enhanced antitumour immune responses. This was attributed to inflammasome activation by OMVs, which led to the release of interleukin-1β (IL-1β). The secreted IL-1β infiltrated the bone marrow and reprogrammed innate immune progenitor cells, thereby enhancing responsiveness to subsequent tumour vaccination [97]. In a related approach, Liang et al. developed a trained immunity-related vaccine (TIrV) based on OMV nanohybrids (OMV-SIRPα@CaP/GM-CSF) that did not rely on tumour-specific antigens. In this system, GM-CSF–loaded OMVs primed bone marrow progenitors and monocytes, which were later recruited to the tumour site by tumour-associated macrophages (TAMs), while SIRPα-Fc–modified OMVs improved TAM-mediated phagocytosis [98]. These studies highlight OMVs as promising tools in cancer vaccine development due to their immunostimulatory properties, stability, low toxicity, and ability to support both adaptive and trained innate immune responses.

### 3.5. Immune Responses to OMVs

It is well acknowledged that multiple primary paths may facilitate the entry of OMVs, including lipid raft-dependent endocytosis, lipid raft-independent endocytosis, macropinocytosis [99], clathrin- [100], and dynamin- [101] and caveolin-dependent entry [99]. Details regarding the process of OMV uptake by cells are reviewed elsewhere [102]. Vanaja et al. provided compelling evidence that LPS is transported into host cells by bacterial OMVs through endocytosis, subsequently releasing LPS into the cytosol to induce caspase-11 activation and inflammatory cytokine secretion [103]. The structure of LPS, particularly the O-antigen region, is essential for OMV entry into host cells. OMVs devoid of O-antigen may utilize clathrin-mediated endocytosis as their primary entrance mechanism; conversely, OMVs possessing complete O-antigens are facilitated by raft-dependent pathways [104]. Additionally, PAMPs displayed on OMVs may initiate TLR signalling to promote the entry of OMVs into host cells. The TLR4 activation contributes to the transfer of LPS into the cytosol by OMVs [105]. It is reported that *Legionella pneumophila* OMV’s membrane merges with eukaryotic membrane systems, potentially transferring pathogenic elements to host cell membranes [106]. In summary, bacterial OMVs possess distinct pathways to enter the host cells and circumvent the epithelial cell barrier, and the characteristics of these OMVs may influence their selection of routes during entry into host cells. Several TLRs of the host cell membrane are stimulated by OMVs. As discussed earlier, TLR4 identifies the lipid A component of LPS [107]. TLR2 with TLR1 and TLR6 detects bacterial lipoproteins, lipoteichoic acids, and peptidoglycan [108]. The identification of flagellin (a protein present in the OMVs of flagellated bacteria) is executed by TLR5 [109], while TLR3, TLR7, TLR8, and TLR9 detect nucleic acids [110]. Unmethylated Cytosine-phosphate–Guanine (CpG) dinucleotides prevalent in bacterial genomic DNA are recognized by intracellular TLR9 [111], as depicted in Figure 3A. OMVs derived from various pathogens contain distinct types and compositions of PAMPs, which can result in varying degrees of reactogenicity. These interactions may elicit the secretion of proinflammatory cytokines. Additionally, innate immune cells primarily neutrophils, dendritic cells, and macrophages in the submucosa identify and engulf OMVs, which initiates an adaptive immune response through antigen presentation, culminating in the activation of CD4^+^ and CD8^+^ T-cells. Overall, the fundamental mechanisms of the adjuvant effects of OMVs involve the activation of TLR4 and the inflammasome, as well as TLR2, TLR9, and TLR5, by the LPS, lipoprotein, CpGs, and flagellin receptively present in the OMVs [107,108,109,112]. A schematic illustration of the interactions between various immune cells and OMVs is given in Figure 3B,C.

## 4. OMV-Based Vaccines

Naturally released outer-membrane vesicles, known as native OMVs (nOMVs), are often low in yield, which is insufficient for vaccine production. To enhance output, OMVs are commonly isolated from whole bacteria using detergents like deoxycholate. This process produces detergent-extracted OMVs (dOMVs). Detergent treatment significantly reduces LPS and lipoprotein levels, lowering reactogenicity and improving tolerability [49,113]. However, this method may also strip away protective lipoprotein antigens, disrupt vesicle structure, and introduce cytoplasmic protein contaminants [41]. Alternatively, genetic modifications can be employed to engineer OMVs with improved characteristics, such as increased yield, reduced toxicity, or tailored protein content. These are referred to as mutant-derived OMVs (mdOMVs) [114]. Generalized Modules for Membrane Antigens (GMMA) are a subclass of mdOMVs. GMMA are designed to hyper-vesiculate, while also incorporating mutations to detoxify lipid A and remove unwanted components, preserving immunogenic surface antigens. In contrast to dOMVs, which can suffer from the unintended removal of surface proteins and disruption of vesicle integrity during processing, GMMA maintain native vesicle structure and antigen composition through genetic modifications that minimize the need for harsh extraction methods. Additionally, GMMA production is more amenable to scale-up, as it avoids the need for chemical extraction steps [114]. Together, these advantages underscore the value of genetic engineering in refining OMV-based vaccine platforms. For a more in-depth comparative evaluation in preclinical contexts, readers are referred to recent reviews and studies [115,116,117,118].

A comparison between nOMVs, dOMVs, and mdOMVs is summarized in Table 2.

### 4.1. Licenced OMV-Based Vaccines

Several OMV-based vaccines have been licenced (Table 3), primarily for protection against *Neisseria meningitidis* serogroup B (MenB) and *Haemophilus influenzae* type b (Hib) infections. Bexsero (GSK), approved in 2013, is formulated with dOMVs from *N. meningitidis* strain NZ98/254, combined with three recombinant proteins (NHBA, NadA, and fHbp). It is recommended for active immunization against invasive MenB disease starting from 2 months of age [124]. Clinical evaluation across 17 studies, including 10 randomized trials, demonstrated acceptable safety and immunogenicity in infants, children, adolescents, and adults. However, a higher incidence of fever (69–79%) was reported when co-administered with routine pediatric vaccines, compared to those receiving routine vaccines alone (44–59%), although most fevers were mild and self-limiting [124]. Following its introduction in the UK, Bexsero led to a 75% reduction in MenB cases among vaccine-eligible infants within three years. Additional studies showed that increasing the OMV dose correlated with higher bactericidal antibody titers, although reactogenicity remained similar across different doses [125]. Coverage analysis using the Meningococcal Antigen Typing System (MATS) indicated a predicted coverage of 81–84% of MenB strains [126].

A similar OMV formulation was deployed in New Zealand to control a MenB outbreak. The vaccine, containing the same OMV as Bexsero but without recombinant proteins, demonstrated strong immunogenicity and an acceptable safety profile in clinical trials. It was administered in a phased national programme from 2004 to 2006, continuing in routine infant vaccination until 2008. The vaccine showed 77% effectiveness and prevented approximately 210 cases of MenB infection between 2004 and 2008 [133,169]. Likewise, in Norway, a dOMV vaccine developed in the 1980s was given to over 170,000 adolescents during 1988–1991, showing an estimated efficacy of 87% after 10 months. However, a decline in protection followed due to waning serum bactericidal activity [170]. This vaccine was later used in France during a localized outbreak of MenB caused by the B14:P1.7,16 strain. Vaccination efforts led to a drop in incidence from 31.6 to 5.9 per 100,000 between 2006 and 2009 [171].

In Cuba, the Finlay Institute developed VA-MENGOC-BC, a dOMV-based vaccine licenced in 1987 to combat MenB. Over the subsequent two decades, the vaccine was associated with a dramatic reduction (93–98%) in MenB disease incidence, effectively eliminating MenB as a public health concern [134,135]. In 2014, VA-MENGOC-BC was acquired by Abivax for distribution in parts of Asia and Latin America.

Hib-OMPC, the first widely licenced OMV-based conjugate vaccine, uses a highly purified capsular polysaccharide from *H. influenzae* type b (polyribosylribitol phosphate, PRP) conjugated to outer-membrane protein complex (OMPC) derived from dOMVs of *N. meningitidis* B11 strain. Licenced as PedvaxHIB (Merck), the vaccine showed 93–100% efficacy in a pivotal trial involving 3486 Navajo infants, a population at high risk of Hib disease. After two doses at 2 and 4 months, 88% of infants achieved anti-PRP titers ≥0.15 μg/mL, and 52% reached ≥1.0 μg/mL, with a geometric mean titer (GMT) of 0.95 μg/mL [129]. A third dose further improved the immune response. PedvaxHIB was found to elicit robust responses, even after a single dose, in high-risk infants [172]. PedvaxHIB was later combined with hepatitis B surface antigen (HBsAg) to create Comvax (marketed as Procomvax in the EU). Although this bivalent vaccine showed slightly reduced anti-PRP antibody levels compared to PedvaxHIB alone, immunogenicity remained sufficient for protection [173]. In five clinical trials involving 1602 children aged from 6 weeks to 15 months, Comvax demonstrated antibody responses comparable to those induced by the monovalent vaccines PedvaxHIB and RECOMBIVAX HB when administered concurrently or at separate sites [174]. The vaccine was indicated for protection against invasive Hib and hepatitis B infections in infants born to HBsAg-negative mothers, following a three-dose schedule at 2, 4, and 12–15 months [174]. Procomvax was voluntarily withdrawn from the EU market in 2009 for commercial reasons unrelated to safety concerns. Later, an OMPC-conjugated Hib antigen was incorporated into the hexavalent pediatric vaccine VAXELIS, which also contains diphtheria and tetanus toxoids, pertussis antigens, HBsAg, and inactivated poliovirus. VAXELIS is administered from 6 weeks of age in a 2–3 dose primary schedule. Safety data were comparable to other pediatric combination vaccines. However, in a booster co-administration study with Prevnar 13 (pneumococcal vaccine), up to 52.5% of infants developed fever, although these episodes were generally mild, having moderate severity, and were resolved within 48 h [175]. Overall, the safety profile of Hib PRP-OMPC vaccines is in line with other approved Hib vaccines, with no clinically significant differences reported [176].

### 4.2. OMV-Based Vaccines in Clinical Development

Many OMV-based vaccines are being explored clinically against various pathogens (Table 3). Generalized modules for membrane antigens (GMMAs), a type of OMV that displays surface O-polysaccharides, are in clinical trials for *Salmonella* and *Shigella* infections [177,178,179,180]. In preclinical models, *Salmonella* Typhimurium and *S.* Enteritidis GMMAs elicited anti-O-polysaccharide IgG levels comparable to CRM197-based conjugate vaccines with alum and demonstrated enhanced isotype diversity and serum bactericidal activity [181]. A bivalent *Salmonella* GMMA vaccine formulated with Alhydrogel is currently in a phase I trial in European adults (NCT05480800) and is also being co-formulated with a *S. typhi* conjugate vaccine (Typhibev) for evaluation in another phase I study (NCT05480800). The first GMMA to enter clinical testing was the *Shigella sonnei* vaccine 1790GAHB, developed using a genetically modified strain (ΔtolR, ΔhtrB) expressing stabilized O-polysaccharides [177]. It has undergone multiple phase I/II trials, including a human challenge model, and was shown to be safe and immunogenic in both endemic and non-endemic populations [137,180,182,183,184]. A booster study conducted three years post-primary vaccination demonstrated a strong anamnestic response [185]. While 1790GAHB did not confer protection in the challenge model, likely due to suboptimal O-polysaccharide dosing, a next-generation version with tenfold higher antigen content was developed. This updated GMMA has been combined with *S. flexneri* 1b, 2a, and 3a GMMAs in a four-valent formulation now in phase I/II trials (NCT05073003) [180]. Additionally, OMV-based *Neisseria meningitidis* B vaccines have shown moderate cross-protection against *Neisseria gonorrhoeae* in epidemiological studies [148]. This has prompted ongoing trials of both *N. meningitidis* B-based vaccines (NCT04297436, NCT04415424) and gonococcal-specific GMMAs (NCT05630859). Heterologous OMV vaccines are also under development. For example, Intravacc BV is evaluating an intranasal OMV-based COVID-19 vaccine candidate, Avacc 10, in early-stage clinical trials (NCT05604690). The iNTS-GMMA vaccine, targeting *Salmonella* Typhimurium and Enteritidis, is based on GMMA engineered to reduce endotoxicity while preserving key surface antigens. It has shown promising safety and immunogenicity profiles in a phase I trial, supporting its potential for preventing invasive non-typhoidal *Salmonella* infections [140,141].

### 4.3. Trends Emerging in Preclinical Studies: Bioengineered/Modified OMV-Based Vaccines

Not all bacteria produce OMVs; therefore, strategies are needed to develop a universal OMV platform for displaying antigens from unrelated pathogens. In addition, OMV cargo differs among strains, which may in certain instances restrict its usefulness to a certain subset of strains. Moreover, numerous strains release minimal quantities of OMVs, indicating that substantial volumes of parent bacteria are required to obtain a good OMV yield. Over the past decade, the bioengineering of bacteria that produce OMVs has significantly advanced to tackle such issues. Recombinant DNA technology significantly expedited the investigation of OMVs in biomedical and other domains. Recently, OMVs have emerged as promising platforms for delivering heterologous antigens [186]. They can be engineered to present proteins or polysaccharides from a variety of pathogens, including viruses, bacteria, and parasites, even those that are phylogenetically unrelated. OMVs can be adorned with heterologous functional components to act not only as antigens, but also as carriers and adjuvants in vaccine formulation.

In summary, bacteria can be engineered to perform the following: (a) overexpress specific homologous vaccine antigens or various forms of a homologous protective antigen; (b) retain and present a vaccine antigen on their surface; (c) eliminate any undesirable antigens that could hinder the development of an effective immune response; (d) display heterologous antigens, whether proteinaceous or polysaccharide, sourced from diverse—including phylogenetically distant—pathogens (viral, bacterial, parasitic); and (e) alter the LPS structure to mitigate associated toxicity [49,187,188]. Genetically altered bacteria-derived OMVs acquire essential genetic components that aid in streamlining the purification process, lowering manufacturing expenses, and, most crucially, guaranteeing natural bioactivity and biocompatibility. Utilizing these alteration options, it can be anticipated to achieve a reasonably designed OMV platform for the intended vaccine.

## 5. Current Strategies and Latest Updates on OMV Bioengineering

**Figure 4:**
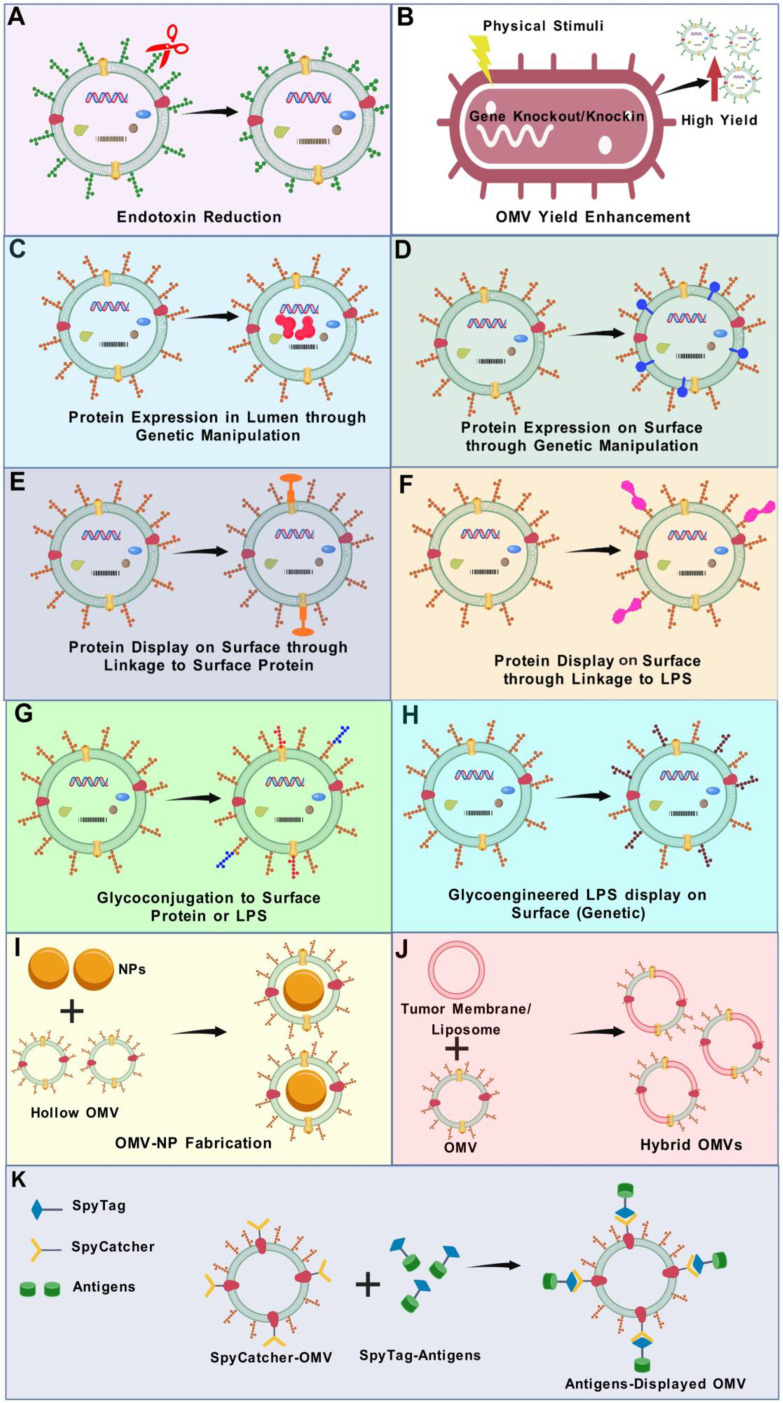
OMVs are a versatile tool for genetic modification and bioengineering in the design of effective vaccines. OMVs can be bioengineered for the following purposes: (**A**) Minimizing the ability of LPS to trigger a reactogenic response following OMV injection [189,190,191]. (**B**) Enhancing the natural ability of Gram-negative bacteria to release OMVs [192]. (**C**) Expressing antigens in the lumen of OMVs [193]. (**D**) Expressing various protein or peptide antigens on the surface through genetically modifying the parent bacteria [186]. (**E**) Linking externally purified protein antigens with the surface proteins of OMVs [194,195]. (**F**) Linking externally purified protein antigens with the surface LPS of OMVs [196]. (**G**) Conjugating polysaccharide-based antigens through fusion with surface proteins or LPS [197,198]. (**H**) Genetically modifying the parent bacteria to express polysaccharide antigens [199]. (**I**) Coating nanoparticles (NPs) with OMVs [200]. (**J**) Preparing hybrid OMVs through the fusion of OMVs with tumour or plant membranes or liposomes [201]. (**K**) SpyTag/SpyCatcher system for antigen display [202]. LPS: lipopolysaccharide; NP: nanoparticles; OMV: outer-membrane vesicle.

In the following section, we will provide an update on various engineering strategies that have been employed to obtain bioengineered OMVs for vaccine development. By leveraging these advanced bioengineering techniques, OMVs have been tailored to enhance immunogenicity, broaden pathogen coverage, and address the challenges associated with traditional vaccine approaches. Figure 4 summarizes the various OMV modification strategies currently employed to bioengineer modified OMVs for vaccine purposes.

### 5.1. Selection of OMV Backbone for Vaccine Development

OMVs inherently possess numerous immunogenic constituents that may elicit a response to the OMV’s backbone. The potential of this effect influences the selection of the OMV’s backbone for vaccine development. The utilization of *E. coli* as a source of the OMV’s backbone has the benefit of substantial prior research [156,199,203,204,205,206]. OMVs utilized in tumour vaccines are mostly sourced from *E. coli* due to their amenability to genetic modification [207,208]. Nonetheless, *E. coli*-derived OMVs pose two primary issues: (i) they are a natural inhabitant of the human body, which raises the possibility of immune tolerance to *E. coli* OMVs, and (ii) robust immune reactions to *E. coli* OMVs may disrupt the normal intestinal flora [209]. The utilization of a pathogenic bacterium for a source of the OMV’s backbone could mitigate these adverse effects. OMVs from pathogenic bacteria, such as *V. cholerae, N. meningitidis*, and *S. enterica* serovar Typhimurium, have been utilized in OMV-based vaccine development [79,159,163], although it remains unclear what impact it has on the dissemination of the producing pathogen itself. The OMV backbone’s cross-reactivity with closely related species has been documented for *N. meningitidis* [210,211], demonstrating the potentially wider impact of immune reactions targeting the OMV’s backbone. The extent to which minimal cross-reactivity affects a vaccination platform or the necessity of a fully stealth OMV backbone remains uncertain. A main consideration should be the potential reaction of the backbone after the repeated use of the same OMV platform for various vaccines.

### 5.2. Reduction in Endotoxin Level

OMVs are effective vaccines and adjuvants because of their PAMPs; nevertheless, PAMPs also cause significant tissue damage by inducing an inflammatory response. The LPS–TLR4 interaction is a primary mechanism of inflammation produced by OMVs. Therefore, mitigating OMV toxicity, especially LPS-associated toxicity, presents a problem for wider applications of OMVs. Multiple strategies are currently in use to acquire OMVs with reduced LPS toxicity.

The first approach involves lowering LPS levels by subjecting pure OMVs to detergents, such as polyoxyethylene 10 oleyl ether (Brij-96) or deoxycholate [49,212]. This technique, however, has the drawback of depleting lipoproteins and enzymes, which act as TLR agonists, hence reducing the adjuvant efficacy of OMVs [121]. Therefore, genetic modifications of OMVs have been suggested to achieve OMVs with enhanced safety and reduced toxicity. In the second strategy, LPS toxicity is lowered by changing the amount of acyl chains or phosphate groups in LPS through genetic manipulations [213]. In *E. coli*, *Shigella*, and *Salmonella*, genes encoding acyltransferases such as MsbB (*lpxM*), htrB (*lpxL*), and pagP responsible for incorporating myristoyl, lauroyl, or palmitoyl groups into lipid A have been knocked out to modify endotoxicity [189,190,191]. Similarly, in *Neisseria meningitidis*, targeted deletions of *lpxL1* and *lpxL2* have been employed for lipid A remodelling [123,214,215]. Comparative studies have shown that OMVs engineered with lipid A mutations, such as *lpxL1* and *lpxL2*, exhibit significantly reduced immunotoxicity. In vitro assays using human PBMCs revealed that Δ*lpxL1* OMVs triggered substantially lower release of inflammatory cytokines (IL-1β, IL-6, IL-8, TNF-α) than wild-type OMVs, comparable to levels seen with dOMVs, despite having higher LPS content [216,217,218]. Similarly, pyrogenicity testing in rabbits showed a dramatic decrease in endotoxic activity, with Δ*lpxL1* OMVs being ~40-fold and Δ*lpxL2* OMVs ~200-fold less pyrogenic than wild-type controls [219]. Phase 1 clinical trials confirmed their favourable safety profile. OMVs with *lpxL2* mutation were well tolerated, inducing bactericidal antibody responses in 12 out of 46 individuals, while Δ*lpxL1* OMVs led to ≥4-fold increases in SBA titers in 79% of recipients, with evidence of cross-strain protection [143,220]. Injection site reactions were generally mild or moderate, and no residual endotoxin-associated effects were observed. These findings support the use of lipid A-modified OMVs as a safer platform for vaccine development. Another study showed that *N. meningitidis* OMVs engineered with penta-acylated lipid A via *lpxL1* or *pagL* mutations significantly reduced IL-6 and IL-1β induction in human immune cells, showing reduced TLR4 activation [221]. Moreover, *E. coli*-derived OMVs engineered to contain only the tetra-acylated lipid IVa variant of LPS demonstrated dramatically reduced toxicity, with pyrogenic activity decreased by 10^5^- to 10^6^-fold compared to OMVs with full-length LPS. Despite this strong attenuation, these OMVs effectively promoted dendritic cell maturation and induced robust, balanced Th1/Th2 immune responses. In animal models, they provided complete protection against heterologous influenza challenges, confirming that the substantial reduction in endotoxicity did not compromise immunogenicity [222]. Kim et al. engineered *E. coli* OMVs with penta-acylated lipid A via *msbB* deletion, resulting in significantly reduced proinflammatory cytokine induction (e.g., IL-6, TNF-α) in vitro. These OMVs maintained immunogenicity while exhibiting lowered endotoxicity, supporting their use as safer vaccine delivery vehicles [223]. Shigella GMMA with lipid A modified by deletion of *msbB* or *htrB* genes exhibited a substantial decrease in toxicity. GMMA from *msbB* mutants showed about a 600-fold drop in TLR4 activation, while those from *htrB* mutants displayed an even greater reduction of approximately 60,000-fold compared to wild-type GMMA. In human PBMCs, these mutants induced inflammatory cytokines at levels reduced by 300-fold and 800-fold, respectively. The remaining immune activation was mainly due to TLR2 signalling, indicating that achieving penta-acylated lipid A is essential to minimize endotoxicity in GMMA-based vaccines [189]. GMMA from *Salmonella Typhimurium* and *Enteritidis* modified by deletion of *msbB* and *pagP* genes produced penta-acylated lipid A and demonstrated reduced cytokine responses and TLR4 stimulation compared to wild-type GMMA [190].

Notably, researchers have progressively begun to investigate other innovative approaches to reduce LPS endotoxicity, for example, utilizing non-pathogenic symbiotic bacteria as OMV sources, which can mitigate the hazards associated with gene deletion and provide a higher safety levels [224]. Moreover, in mice, pre-treatment with all-trans retinoic acid (ATRA) before administrating *V. cholerae* OMVs has been shown to reduce toxicity by downregulating TLR2 responses [225]. Nie et al. presented a novel approach to mitigate OMV toxicity and augment OMV accumulation within tumours while simultaneously delivering tumour antigens through the chemodynamic impact which induces immunogenic cell death (ICD). The interfacial coordination between tannic acid (TA), a natural polyphenol approved by the Food and Drug Administration (FDA), and ferric ions (Fe^3+^) promotes the formation of a metal-phenolic network (MPP) on the surface of OMVs, effectively creating a stable protective layer [226]. Following systematic dosing, MPP reduces systemic toxicity and increases the OMV accumulation within tumour. The breaking of coordination bonds between TA and Fe^3+^, prompted by the acidic pH and elevated ATP levels in tumour microenvironment, results in OMV release that reprograms macrophages associated with tumour to switch to the tumouricidal M1 phenotype from the pro-tumoural M2 phenotype. As a result, the levels of hydrogen peroxide (H_2_O_2_) and proinflammatory molecules rise, leading to enhanced ICD. In contrast, OMVs, acting as potent adjuvants, engage with DMAPs generated through the ICD cascade and tumour antigens, and subsequently elicit a vigorous anticancer immune response [226].

Beyond endotoxicity, concerns such as unintended immune activation, the risk of autoimmunity, and long-term immunological consequences also warrant careful consideration. A comprehensive evaluation of these issues is beyond the scope of this review. However, readers interested in a more detailed analysis of OMV safety are referred to other reviews [223,227,228].

### 5.3. Strategies to Enhance OMV Production

In industrial vaccine production, it is essential to develop ways for enhancing OMV yields to optimize production volume and reduce costs. Under typical conditions, OMV release is predominantly governed by growth stages, which affect OMV production in both quantity and quality [192]. An ideal harvest period, often during the late exponential phase, is essential to maximize the yield of OMVs while preventing contamination caused by bacterial cell lysis following prolonged incubation [9]. Consequently, supplementary approaches to enhance OMV yield, encompassing chemical, genetic, and physical mechanisms, have been explored. Nutrient shortage, temperature fluctuations, and the addition of phages and antibiotics may all lead to an increased OMV yield (reviewed in detail elsewhere [1,192]). For example, a recent study demonstrated that the *Acinetobacter baumannii* phage lysin, LysP53, enhances OMV production upon interacting with *A. baumannii*, *E. coli*, and *Salmonella*. The lysin-derived OMVs (LOMVs) from *A. baumannii* exhibited greater uniformity, reduced endotoxin levels, higher protein yield, and lower cytotoxicity compared to naturally produced OMVs [229]. The secretion of OMVs can be modulated by the introduction of particular organic or inorganic compounds to bacterial cultures, either by directly stimulating the bacteria to enhance vesiculation process or by generating environmental stress that leads to enhanced vesiculation. Detergent-based extraction can also increase OMV yield. Sonication is a widespread physical technique utilized for the generation of OMVs, leading to the fragmentation of bacterial cells that subsequently aggregate into synthetic OMVs. A primary concern with this technology is that the composition of OMVs may significantly differ from spontaneously released OMVs. For example, cytoplasmic proteins were predominantly present in protein profiles from chemically manufactured OMVs of *Glaesserella parasuis*, but outer-membrane and periplasmic proteins were predominant in spontaneously released OMVs [230].

Li et al. employed a high-pressure homogenization technology that resulted in a sixfold higher OMV yield in *E. coli* [231]. To keep the structure of OMVs stable, the wall-breaking phenomenon can be regulated. This approach may be utilized to improve the generation of OMVs in different bacteria. Metal chelators constitute a distinct category of compounds that can enhance OMV yield by removing metal ions from the solution, hence establishing restrictive growth parameters that frequently lead to heightened bacterial vesiculation. Ultimately, environmental stressors like oxidative stress can be elicited by the introduction of hydrogen peroxide or the reduction in cysteine, prompting survival mechanisms and hypervesiculation in specific bacteria [232,233,234].

Gene knockout is a well-recognized technique for the targeted inactivation of particular genes. By targeting crucial genes that are either directly or indirectly involved in the biogenesis of OMVs, yield can be enhanced. The biogenesis of OMVs encompasses critical events, including alterations in fluidity and curvature of the membrane, disruption of crosslinks joining peptidoglycan and outer membrane, and the aggregation of misfolded proteins. The processes governing these links can be exploited to influence the generation of OMVs. Genes associated with the crosslinking of outer membrane with peptidoglycan, including Lpp, nlpI, the Tol-Pal system, and peptidoglycan-degrading enzymes, can be mutated to enhance the generation of OMVs [231,235,236,237,238,239,240,241]. The perturbation of the VacJ/Yrb ABC (ATP-binding cassette) transport system was successfully employed to enhance OMV synthesis in *E. coli, H. influenzae,* and *V. cholerae*. Phospholipid buildup in the outer membrane is thought to be the cause of this increased OMV yield [37,242]. Deletion of RmpM [119,243], MltA (gna33) [40], OmpT [244], enterobacterial common antigen [242], PagL [245], Virk [246], and DegP [247] has also been shown to induce excessive vesiculation. Moreover, mutations in genes that enhance membrane curvature, including phospholipase A and CL, can elevate the synthesis of OMVs [32,248]. OMVs produced by genetically engineered hypervesiculating bacteria are also known as generalized modules for membrane antigens (GMMA) [123,249].

### 5.4. Lumen Expression of Heterologous Antigens

#### 5.4.1. Endogenous Loading

Overexpression of antigens can direct them to the periplasmic area, where the OMV’s lumen will encapsulate and store them [78]. For example, multiple Streptococcus proteins were conjugated with the signal peptide of outer membrane protein A (OmpA) of *E. coli*, and, upon reaching the periplasm, they were effectively encapsulated into OMVs [203]. These modified OMVs elicited elevated functional antibody titers against the recombinant version of the proteins in mice [203]. A comparable method was utilized to produce *E. coli* OMVs containing the *Chlamydia muridarum* antigen HtrA [147]. Mice vaccinated with modified OMVs of *Salmonella* expressing pneumococcal protein PspA (pneumococcal surface adhesin A) in the OMV’s lumen elicited significant titers of serum antibodies against PspA, as well as responses against bacterial LPS and outer membrane proteins. Mice inoculated with an identical amount of recombinant PspA had no measurable antibody responses to PspA [78]. These findings hold significant therapeutic implications, as PspA is a conserved protein among pneumococcal serotypes and a strong immune response to PspA may protect from several serotypes. A separate study demonstrated the delivery of F1V antigen from *Yersinia pestis* fusion protein into the lumen of OMVs via the OmpA leader sequence, leading to improved protective efficacy against *Y. pestis* in mice vaccinated with these OMVs [193]. Although the proteins in the above examples were not exposed on the surface yet, they successfully induced antigen-specific antibodies [78,147,203]. The produced IgG antibodies exhibited functional efficacy regarding opsonophagocytosis of bacteria and the protection of mice from lethal infection challenge [78]. This can be explained by a proposed mechanism in which OMVs are taken up by antigen-presenting cells (APCs), allowing for intracellular processing and presentation of luminal antigens via MHC molecules to T-cells. Concurrently, a fraction of OMVs may undergo partial degradation at the injection site, exposing internal proteins to naïve B-cells for direct recognition. The activation of these B-cells is then supported by T-cell help, collectively contributing to a strong antibody response [203].

#### 5.4.2. Exogenous Loading

The incorporation of antigens into the lumen of OMVs, following the mass production of both vesicles and antigens, can be a compelling strategy. With this approach, the regulation of active component concentration may be more efficient than that of endogenous loading. Gujrati et al. employed electroporation for encapsulating siRNA into OMVs derived from *E. coli* [94]. The siRNA used in this study targeted the kinesin spindle protein, which is overexpressed in tumours and rapidly developing cells. The loaded vesicle was directed to HER2^+^ tumour cells via a HER2 antibody conjugated to ClyA on the OMV’s surface [94]. The vesicle must be opened and then resealed without bearing permanent damage in order to introduce intact antigens into the OMV’s lumen. At present, there are no reports of the exogenous introduction of proteins into the OMV’s lumen, whereas many techniques for incorporating small molecules into EVs are being investigated. Future research may confer more insight into this field.

Few studies have directly compared antigen localization in OMVs, whether on the surface or enclosed within the lumen. Clear evidence favouring surface localization comes from *N. meningitidis* OMVs displaying OspA, which generated robust anti-OspA responses, unlike their luminal counterparts [159]. Likewise, *S. typhimurium* GMMAs with surface-conjugated fHbp induced significantly stronger immune and bactericidal responses than when the same antigen was enclosed within the lumen or physically mixed with GMMAs [250]. On the other hand, for all surface-exposed antigens, there are strong evidence of antibody responses. Therefore, displaying proteins on the surface of OMVs is generally favoured to achieve a more robust immune response.

### 5.5. Modifications of OMVs in Terms of Surface Display of Heterologous Antigens

To improve and broaden the vaccine potential of OMVs, synthetic biology and recombinant DNA technology approaches are being employed to design OMVs with heterologous peptide, protein or polysaccharide cargo on their surface. Antigens that are expressed on the surface of OMVs are available for binding by antigen-specific B-cells, making them preferred in the design of modified OMVs. The physical attachment of antigens with OMVs, rather than mere mixing of unattached antigens with OMVs, is essential for generating robust immune responses to diverse antigens [114,202,251]. Generally, the genetic coupling of the peptide or protein to a scaffold protein in the outer membrane results in the accumulation of the peptide/protein of interest in released OMVs, which can be readily extracted from the culture supernatant.

In recent years, various strategies have been employed for surface display of heterologous antigens, which are discussed below.

#### 5.5.1. ClyA Fusion

Cytosolic A (ClyA) is a cytosolic protein of 34 kDa encoded by the *ClyA* gene, alternatively referred to as *hlyE* and *sheA*, found on the K-12 chromosome of *E. coli* [252]. ClyA functions as a signal sequence, facilitating the outer-membrane localization of heterologous proteins from where OMVs are released, hence enabling the presentation of protein antigens on OMV surfaces [253]. Fusing the target protein with the N-terminal of ClyA results in variable expression of antigens, whereas fusion with its C-terminal consistently produces proteins that are well-expressed and have proper biological activity [253]. This may be attributed to the close proximity of the C-terminus to the outer surface of the membrane, which can enable its extension into the extracellular space [254,255]. Numerous studies have used *E. coli* ClyA as the fusion partner for the delivery of foreign proteins to OMV surfaces. For instance, ClyA has been conjugated with the ectodomain of the influenza A matrix protein 2 (M2), green fluorescent protein (GFP), and the domain 4 component of the *B. anthracis* protective antigen [156,256,257]. 

#### 5.5.2. Ice Nucleation Protein (INP) Fusion

The ice-nucleation protein (INP) is naturally present in bacteria capable of promoting ice formation [258]. Its N-terminal region functions as a membrane anchor, facilitating the extracellular presentation of the C-terminal region. Additionally, proteins attached to either or both ends of INP are displayed on the bacterial surface. Notably, INP can present the trivalent scaffold Scaf3 on the bacterial surface through the formation of an INP-Scaf3 fusion [63,259]. Similarly to the AT system, this enzyme scaffold serves as a high-efficiency platform that can concurrently load numerous heterologous proteins, creating a multifunctional system autonomously. It also organizes target proteins in a structured fashion, creating a multifunctional platform on its own [260]. Nonetheless, the INP system has not yet been explored for displaying vaccine antigens on the surface of OMVs. It will be interesting to see the use of this platform for displaying heterologous antigens on OMV surfaces.

#### 5.5.3. OMV Decoration Using Lipoprotein (Lpp) Transport Machinery

Lpp (Braun’s lipoprotein) is a prevalent lipoprotein found in the outer membrane of bacteria. Cowles et al. reported that the free-form Lpp in *E. coli* has the ability to transport the FLAG epitope to the cell surface [261]. The expression of heterologous antigens using the Lpp signal sequence is significantly higher compared to when the same antigens are produced within the OMV’s lumen through periplasmic delivery using the bla-SS signal sequence. This observation is believed to stem from the extensive localization of Lpp in the periplasm and outer membrane of Gram-negative bacteria [186]. Thus, the lipidated fusion antigen, carrying a lipoprotein leader sequence, may displace part of the space normally taken up by Lpp in the outer membrane and periplasm of bacteria. This would likely lead to an enhanced distribution of heterologous antigens on both the OMV’s surface and within its lumen. Lipidating heterologous antigens offers an immunological benefit, as lipoproteins are powerful activators of the immune system, stimulating the host’s innate immunity and promoting a robust adaptive immune response [262]. Irene et al. utilized the Lpp transport system to express five protective antigens of *S. aureus* on *E. coli* OMVs. These antigens produced in *E. coli* as fusions to the Lpp sequence. Lipidated antigens not only accumulated in the vesicular compartment in higher quantities, but also disrupted the acylation of lipid A, thus diminishing the LPS-mediated reactogenicity. Animals inoculated with modified OMVs exhibited full protection against *S. aureus* challenge [263]. Another recent study developed modified OMVs utilizing the Lpp transport system to display the SaoA antigen from *Streptococcus suis*. The findings of this study showed that Lpp-SaoA fusions can be presented on the OMV’s surface without exhibiting considerable toxicity. Additionally, they can be concentrated in OMVs, constituting almost 10% of the total OMV proteins. Immunization with OMVs incorporating the Lpp-SaoA fusion antigen elicited a robust antibody response and elevated cytokine levels, alongside a balanced Th1/Th2 immune response [186]. Moreover, the vaccine markedly improved pathogen clearance in a murine model of *S. suis* infection [186]. The findings of these experiments present a promising and adaptable approach for the development of modified OMVs and indicate that Lpp-based OMVs could serve as a ubiquitous vaccine platform for major pathogens.

#### 5.5.4. The Hemoglobin Protease (Hbp) Display System

The hemoglobin protease (Hbp), a virulence factor of *E. coli*, has been adapted for use in a surface display system, enabling its high-density expression on live *S. enterica* serovar Typhimurium and *E. coli* cells, as well as on their OMVs. This system allows for the genetic incorporation of multiple antigenic sequences within the Hbp core, thereby enhancing their recognition by the immune system. The *E. coli* Hbp has been employed to present *Mycobacterium tuberculosis* antigens and *Chlamydia trachomatis* MOMP epitopes on *Salmonella typhimurium* OMVs [163,264]. The Hbp-based platform was also employed to present two *Streptococcus pneumoniae* protein antigens on *Salmonella*-derived OMVs. Intranasal administration of these OMVs, which displayed increased levels of the antigens, conferred significant protection in a murine model of pneumococcal colonization, even in the absence of a mucosal adjuvant [265]. The presence of the Hbp function in the passenger side domains ensures that substituting them with antigenic proteins inherently mitigates possible deleterious consequences, rendering the proposed Hbp platform secure for antigen delivery [266]. Despite the Hbp display platform’s relative tolerance, augmenting the quantity, complexity, and size of integrated sequences typically diminishes the expression of the fused constructs and constrains the display density [163,265]. This outcome is attributed to the intricate mechanism of Hbp secretion through the outer membrane, along with the efficient quality control system that retains translocation-deficient chimeric Hbp molecules within the periplasm. A potential solution to this challenge involves fragmenting the antigen and strategically incorporating the resulting segments into multiple permissive integration sites within the Hbp structure [163,265,267]. Nevertheless, this approach carries the risk of disrupting conformational epitopes, which may affect antigen recognition and immune response efficacy. Given that the antigen’s density on the OMV’s surface is crucial for protective efficacy, alternative approaches are essential.

To overcome this challenge, purified proteins are conjugated to the Hbp carrier post-translocation across the outer membrane, utilizing the newly developed SpyTag/SpyCatcher protein ligation system, which will be discussed in the following section.

#### 5.5.5. Spy Tag (SpT)/Spy Catcher (SpC) System

Many antigens that are advantageous for vaccine development are unsuitable for expression on the surface or in the lumen of OMVs using bacteria as an expression host. The primary bottlenecks often involve proteolytic degradation, misfolding, incorrect or absent post-translational modifications, and ineffective bilayer translocation of the protein of interest—particularly for proteins that are large and/or structurally complex. The absence of effective methods to predict the expression potential of OMV-directed antigens renders the development of heterologous OMV vaccines a laborious trial-and-error process, often requiring repetition for each novel antigen. Furthermore, it is difficult to precisely control the amount of antigen associated with OMVs, making antigen density an inflexible feature in vaccine design. While integration of polysaccharide or polypeptide production with vesiculation has been demonstrated [113,268], the biosynthesis of other types of biomolecules remains unproven, potentially limiting OMV’s cargo diversity.

To overcome these limitations, modular strategies for OMV functionalization have been developed, allowing for OMV vectors and structurally diverse antigens to be produced separately and then conjugated in a controlled manner. Although direct chemical conjugation of polysaccharides and proteins to OMVs post-purification has been reported [251]. Such approaches involve non-specific binding to undefined OMV components, resulting in heterogeneity, reduced immunogenicity, and difficulty in prediction or analysis. To bypass translocation bottlenecks, antigens can be covalently attached to pre-existing surface-exposed OMV components. The SpyCatcher/SpyTag system was developed to enable site-specific covalent conjugation of purified antigens to Hbp displayed on OMVs [269]. This protein ligation system is derived from the fibronectin-binding protein FbaB of *Streptococcus pyogenes*, where an internal isopeptide bond forms autocatalytically between a lysine and an aspartic acid residue. By splitting the domain into a 13-amino-acid SpyTag and a 138-amino-acid SpyCatcher, an intermolecular isopeptide bond can be formed under a wide range of buffer, temperature, and pH conditions [269]. This modular “click” system enables rapid and stable attachment of multiple antigens to OMVs in vitro, creating flexible multi-target vaccine platforms. Importantly, the conjugation of purified proteins to Hbp after its membrane translocation does not impair their surface presentation on OMVs. The integration of the SpyCatcher/SpyTag system has significantly enhanced OMVs as nanocarriers for recombinant proteins and tumour antigens [194,202,270]. This allows for multiple proteins to be coupled to a single Hbp or OMV scaffold, enabling the production of multivalent OMVs in a single batch, eliminating the need for parallel expression systems and avoiding instability from co-expressing multiple constructs that may undergo homologous recombination. Additionally, antigens can be produced in appropriate expression systems to preserve their native conformation and function [202]. The SpyTag/SpyCatcher system has been used to display single, bivalent, or multivalent antigen combinations on OMVs [194,270]. A key example is the work by Sun et al., who displayed multiple antigens from *Staphylococcus aureus* on *E. coli* MG1655-derived OMVs using OmpA-SpyCatcher fusions, creating a multitarget “click” vaccine. Compared to antigens formulated with alum adjuvant, the click vaccine elicited stronger antigen-specific humoral and Th1-biased cellular immune responses, providing protection against lethal *S. aureus* challenges in a murine model [271].

#### 5.5.6. SnoopTag (SnT)/SnoopCatcher (SnC) System

A recently reported protein ligation system based on the *S. pneumoniae* adhesin RrgA is termed the SnoopTag/SnoopCatcher system [272], in which multiple antigen modules could be coupled to Hbp in a sequential ligation strategy [270]. It is developed by dividing the SpyCatcher into the SpyLigase domain and the 10-amino acid peptide KTag (KT) [273]. The SpyTag and KTag possess amino acid residues Asp and Lys, respectively, that constitute the isopeptide bond, whilst the catalytic activity is encompassed inside the SpyLigase domain, which can be incorporated independently. Thus, this OMV coupling approach demonstrates versatility and robustness, facilitating the rapid generation of vaccines via a modular plug-and-display method [270]. For example, Cheng et al. developed a tumour OMV vaccine platform using a SpyTag/SpyCatcher pair and a SnoopTag/SnoopCatcher pair to attach protein tags with protein capture elements through isopeptide bonds to quickly display tumour antigens. Immunization with this vaccine induced tumour-specific T-cell production [202].

It is noteworthy that the potential for using alternative carrier proteins from less-studied bacterial species remains unexplored. Examples of such alternative carrier systems include ApfA and fHbp, which are employed for antigen membrane enrichment in *Actinobacillus pleuropneumoniae* and *N. meningitidis*, respectively [159,274].

#### 5.5.7. Display of Biotinylated Antigens on OMV Surfaces via the AvidVax Platform

Site-specific conjugation approaches are desirable for more accurate and homogeneous antigen attachment. As mentioned previously, the SpyTag/SpyCatcher protein ligation system enables the covalent attachment of purified SpyTag-antigen (or SpyCatcher-antigen) fusion proteins to corresponding SpyCatcher-scaffold (or SpyTag-scaffold) fusions, which are expressed on the OMV’s surface. Although this approach enables the incorporation of foreign antigens onto OMVs [202,270], it is limited to antigens that can form isopeptide bonds. A recently reported avidin-based vaccine antigen crosslinking technology called AvidVax can address these challenges by attaching biotinylated antigens on the OMV’s surface. This strategy allows for a faster and streamlined assembly of antigens onto the surfaces of OMVs. In this technology, the OMVs are altered to transport a synthetic antigen-binding protein (SNAP), consisting of an outer membrane scaffold protein and a biotin-binding protein. The resultant SNAPs facilitate the effective adornment of OMVs with a molecularly varied assortment of biotinylated antigens, encompassing membrane proteins, globular proteins, glycoconjugates, glycans, short peptides, lipids, haptens, and nucleic acids. SNAP-OMVs, decorated with antigens, induce a robust antigen-specific antibody response that is superior to that observed with traditionally manufactured OMV formulations [195,275]. In summary, AvidVax is a flexible and customizable platform for vaccine development, enabling rapid cycles of synthesis, testing, and production of antigen-loaded OMVs for use as vaccines against various pathogens.

#### 5.5.8. OMV Surface Decoration by Molecular Painting (MP)

Molecular Painting (MP) is associated with glycosylphosphatidylinositol (GPI) anchoring. GPI anchors consist of a carbohydrate core rich in mannose residues, paired with a phosphoinositol group that links the anchor to fatty acid chains (either acyl or aryl), which become integrated into the outer membrane’s lipid layer. The protein is connected to the anchor through an ethanolamine bond. The specific sequence guiding proteins to undergo GPI anchoring is referred to as the GPI signalling sequence (GSS), which enables the incorporation of foreign proteins when included in expression systems [276,277]. Recently, Zaruba et al. extended the application of MP to include bacterial OMV surfaces, successfully attaching proteins synthesized in eukaryotic cells to OMVs, thereby generating genuine prokaryotic/eukaryotic hybrid structures. The modified OMVs retain their adjuvant properties, offering a method to quickly develop vaccine formulations with improved safety. This approach allows for the incorporation of various protein factors during the process. As the antigenic structure originates from eukaryotic sources, it carries specific eukaryotic glycosylation patterns and post-translational modifications (especially human-specific if human cell lines are used for GPI-anchored protein production), providing an advantage in stimulating specific immune responses [278]. Through MP, two different GPI proteins can be displayed on the same OMV [278]. This technology has demonstrated the transfer of antigens, fluorescent marker proteins, growth factors, and cytokine on OMV surfaces, creating a versatile modular platform for an innovative vaccine approach.

#### 5.5.9. LPS-Binding Peptides for the Display of Antigens on OMV Surfaces

A subunit COVID-19 vaccine, composed of a D614G mutation stabilized SARS-CoV-2 spike protein fused to the LPS-binding peptide mCRAMP from meningococcal OMVs, induced strong neutralizing antibody and mucosal responses following intranasal administration in the Syrian hamster model. The candidate also conferred protection in a viral challenge study [196]. mCRAMP, and its human orthologue LL-37, are cathelicidin-derived host defence peptides that bind LPS via electrostatic interactions with the phosphate groups of lipid A and the inner core oligosaccharide, leading to membrane insertion through their amphipathic structure [279]. This LPS-binding property can be utilized to direct a fusion protein to associate with LPS on the OMV’s surface. This method allows for independent expression of recombinant antigens using suitable expression system and then subsequent attachment to OMVs. Moreover, mCRAMP and LL-37 have natural immunomodulatory properties that may enhance mucosal immune responses [279]. Further validation with other antigens and pathogens is needed to confirm its broader applicability.

#### 5.5.10. Chemical Conjugation of Antigens to OMVs

Advanced chemical conjugation techniques facilitate the attachment of various molecules, such as new antigens, adjuvants, or targeting ligands, to OMVs’ surfaces while preserving their structural integrity. To a certain extent, chemical conjugation allows for control over the quantity and density of antigens on OMV surfaces [114]. Chemical conjugation requires the attachment of target antigens to the OMV’s surface via covalent bonds. The most commonly used functional groups for chemical conjugation comprise carboxyl groups (-COOH), amino groups (-NH_2_), thiol groups (-SH), and azide groups (-N_3_). Moreover, the introduction of cross-linking agents can aid in the creation of stable covalent links. Such reagents contain functional groups that interact with the functional groups on the OMV’s surface. For example, amine-reactive crosslinkers, like N-hydroxy succinimide (NHS) esters, facilitate the formation of stable amide linkages with the amine groups present on the OMV’s surface [280].

Micoli et al. employed chemical conjugation to link a variety of heterologous OMV-antigen models, such as MenB fHbp or malaria protein antigens with *Salmonella* typhimurium GMMA, ETEC proteins with *Shigella sonnei* GMMA, MenA or MenC oligosaccharides with MenB or *Salmonella* typhimurium GMMA, and *H. influenzae* oligosaccharides with MenB GMMA. The chemical conjugation process did not negatively affect the GMMA-specific humoral immune response in terms of antibody production (e.g., evaluated through anti-LPS response measurement) or functionality, such as bactericidal activity [114]. *Brucella abortus* LPS was attached to *N. meningitidis* serogroup B OMVs, utilizing carbodiimide for coupling and adipic acid for linking the LPS to the OMVs [197]. An optimized conjugate meningococcal vaccine targeting B and C serogroups comprised serogroup C CPSs conjugated to OMVs from serogroup B. This conjugation employed adipic acid dihydrazide as a spacer and EDAC (1-ethyl-3-(3-dimethylaminopropyl)carbodi-imide) as a catalyst, focusing on enhancing synthesis yield, while preserving the antigenicity of both constituents [281]. Di Benedetto et al. outlined the development of two simple and versatile conjugation strategies designed to efficiently attach antigens to the OMV’s surface. These methods aim to reduce the number of conjugation steps, avoid the need for protein antigen modification, ensure high conjugation efficiency, and enable targeting of various OMV components, including proteins or LPS/lipooligosaccharide (LOS). A Design of Experiment methodology was employed to determine the best conditions for OMV activation prior to conjugation, yielding a reliable conjugation procedure irrespective of the conjugation antigen. The application of orthogonal chemistries, aimed at distinct components on OMVs, enabled the precise attachment of two separate antigens on a single OMV particle, facilitating the advancement of multivalent vaccines addressing multiple diseases concurrently. The conjugation process begins with the functionalization of GMMA proteins using the divalent homobifunctional linker Bis(sulfosuccinimidyl) suberate (BS^3^), followed by the interaction between the GMMA–linker complex and the foreign protein antigen [282]. The chemical method has only recently been applied to viral antigens such as influenza A virus hemagglutinin and rabies glycoprotein via BS^3^ chemistry, demonstrating that OMVs can markedly enhance both antigen-specific humoral and cellular immune responses [115].

In conclusion, chemical conjugation enables the attachment of antigens regardless of their origin, size, or posttranslational state using a uniform chemical reaction, facilitating straightforward antigen replacement for various vaccines. However, issues like steric hindrance, linker toxicity, and limitations in antigen loading capacity [283] need to be addressed to fully harness the potential of chemically engineered OMVs.

#### 5.5.11. Engineering Display of mRNA Antigens on OMV Surfaces

The successful immune activation driven by mRNA-based vaccination hinges on its capacity to effectively engage APCs, which serves as a crucial prerequisite for its efficacy [284]. Nonetheless, owing to its inadequate stability, pronounced negative charge, and substantial molecular weight, an mRNA vaccine necessitates effective delivery vehicles to penetrate cells [285]. Lipid nanoparticles remain the leading carriers for in vivo clinical mRNA delivery, encapsulating mRNA within nanocarriers using a microfluidic-based manufacturing strategy [286]. The complexity and variability of tumour antigens render this protracted encapsulation method unsuitable for the tailored manufacture of a personalized tumour vaccine [287]. Moreover, in order to activate the adaptive immune response effectively, support of the innate immune system is required; hence, mRNA vaccines typically need the concurrent delivery of an immunological adjuvant, which complicates the manufacturing process [288]. Consequently, there is an urgent need for a nanocarrier capable of swiftly presenting mRNA antigens while also stimulating innate immunity to advance the progress of mRNA-based tailored tumour vaccines. Nonetheless, the application of an OMV-based platform as a delivery system for mRNA vaccines remains largely unexplored, with effective strategies for displaying mRNA-encoded antigens still facing significant limitations. A study employed RNA-binding protein technology to functionalize OMVs, enabling the integration of mRNA antigens on OMVs. To achieve this, the archaeal RNA-binding protein L7Ae was fused to the C-terminal region of the OMV surface protein ClyA. Meanwhile, a specific RNA-binding sequence, box C/D, was incorporated into the 3′-untranslated region (UTR) of the in vitro-transcribed mRNA. This box C/D sequence adopts a characteristic k-turn structure, which is specifically recognized by L7Ae, facilitating stem-loop stabilization and forming a distinct L7Ae-k-turn complex [289,290]. The robust and highly specific interaction between the box C/D sequence and L7Ae enabled the rapid attachment of box C/D-labelled mRNA (box C/D-mRNA) to the OMV’s surface. To improve endosomal escape and promote efficient mRNA translation, listeriolysin O (LLO) was fused to the C-terminal region of OMV-associated ClyA. The engineered OMV-based vector efficiently displays mRNA antigens and ensures their targeted delivery into dendritic cells, enabling optimal translation, antigen processing, and presentation. The presence of PAMPs in OMVs stimulated innate immune responses, leading to the activation of antigen-specific T-cells, significantly suppressing tumour growth in mice models of subcutaneous colon cancer and lung metastatic melanoma. Thus, this platform introduces a novel nanocarrier for mRNA delivery alongside an innovative antigen-loading strategy, paving the way for its broad application in personalized tumour vaccines and other vaccine developments [291]. Li et al. evaluated the efficacy of mRNA-decorated OMVs in melanoma and colon cancer models, demonstrating promising outcomes [292]. Therefore, the effective use of OMVs can lead to the fast development of mRNA vaccines targeting complex and heterogeneous antigens.

### 5.6. Strategies to Improve OMV Vaccines’ Cross Protection

The immune protection provided against various strains is typically thought to be somewhat restricted because of the differences in the immune-dominant antigens present on the surface, which can vary significantly [293]. This is also applicable for OMV-based vaccines, where the immunogenic properties of different OMV preparations depends upon the profile of the OMPs. Immunization with wild-type OMVs elicit antibodies to immunodominant antigens. If these immunodominant antigens express a high level of variations among different strains, then immunization with such antigen-containing OMVs will lead to limited bactericidal activity against strains having variants of those immunodominant proteins. Furthermore, these immunodominant proteins also make other proteins present in the outer membrane under-represented or even absent for the host immunity [294]. These observations suggest that modifying the outer-membrane protein profile of OMVs can influence their antigenic and immunogenic properties, potentially enhancing cross-protection. Indeed, *N. meningitidis* OMVs derived from PorA-, PorB-, and RmpM-deficient strains had broad cross-reactive bactericidal antibodies, killing a wide range of heterologous strains [295]. Moreover, the deletion of specific small non-coding RNAs has been shown to improve the protective potential of *H. pylori* OMVs in murine models [296]. Similarly, immunization via intranasal or intraperitoneal routes using *Salmonella typhimurium* OMVs lacking flagellin conferred effective cross-protection against *S. choleraesuis* and *S. enteritidis* [297]. Additionally, OMVs derived from *S. typhimurium* mutants deficient in major outer-membrane proteins demonstrated improved heterologous protection [298]. Lietner et al. aimed to develop a serogroup-independent OMV-based cholera vaccine by removing the O antigen, the primary distinguishing feature between serogroups O1 and O139, with the goal of redirecting the immune response to shared OMV antigens. Using OMVs derived from a ΔwaaL ΔmsbB strain lacking the O antigen and containing underacylated LPS, they found that immunization did not provide protection against either serogroup. These results indicate that antibodies against the O antigen are critical for protective immunity against *V. cholerae* [299]. Therefore, it is not always feasible to improve cross-protection by removing strain-variable immunodominant antigens.

Pooling OMVs from multiple bacterial strains has emerged as a promising strategy to enhance the breadth of immune protection offered by OMV-based vaccines. Combining OMVs with distinct antigenic profiles can broaden the immune response, enabling the recognition and neutralization of diverse pathogen variants. For example, in vaccine development against avian pathogenic *E. coli* (APEC), multi-serogroup OMVs (MOMVs) were generated by mixing OMVs from three different APEC strains. This approach aimed to address the challenge posed by the numerous serogroups of APEC, which complicate vaccine design. The MOMVs demonstrated the ability to induce innate immune responses and provided cross-protection against various APEC infections in broiler chickens [300]. Similarly, a tetravalent OMV-based vaccine was developed against *Shigella* by using the most prevalent strains of *Shigella* by pooling OMVs from each bacterium in equal proportions [301].

### 5.7. Glycoconjugated and Glycoengineered OMVs

Vaccines composed only of polysaccharides/oligosaccharides generally elicit T-cell-independent immune responses, lacking IgM to IgG class switching and failing to establish immunological memory [302]. A prevalent approach to induce immunological memory is the covalent conjugation of the polysaccharide to a carrier protein. Nonetheless, the existing technology for the production of conjugate vaccines necessitates intricate synthetic chemistry for the acquisition, activation, and conjugation of polysaccharides/oligosaccharides to a protein carrier. The chemical processes required for glycan purification and protein crosslinking can compromise certain glycans containing acid-labile sugars, making them susceptible to degradation. Notably, chemical crosslinking frequently lacks reproducibility, leading to inconsistent results characterized by batch-to-batch variability. Recent progress in bacterial glycoengineering has enabled the decoration of OMV surfaces with non-native polysaccharide antigens. This innovation has given rise to a new class of glycoconjugate vaccines that effectively display pathogen-like glycan epitopes, stimulating the immune system and offering protection against future infections. Glycans of interest, such as O-antigens, CPSs, or eukaryotic N- or O-glycans, can be displayed on the surface of OMVs [303]. Given their simple production and purification processes, along with their potential to enhance immune responses, OMVs have recently been widely explored as carriers for polysaccharides [114,116,181,198,304,305]. OMVs can act as carriers for chemically linked polysaccharides, allowing for conjugation either to LPS/LOS or to surface-exposed proteins on the vesicles [116]. Various structurally distinct polysaccharides from pathogens, such as *N. meningitidis* (serogroups A and C), *H. influenzae* type b, *Streptococcus* group A, and *Salmonella typhi* Vi, have been successfully covalently attached to GMMAs, eliciting strong anti-polysaccharide immune responses in animal models [114,198,305]. Antibody levels and function were largely unaffected by the number of glycans per OMV, although lower glycan loading better preserved the immunogenicity of OMV proteins. Glycan length required case-specific optimization, and conjugation to LOS/LPS was as effective as protein linkage in inducing strong immune responses [116].

Beyond chemical conjugation, OMVs can be genetically modified to express heterologous glycans, creating what are known as glycoengineered OMVs (geOMVs). *E. coli* strains lacking native O-polysaccharide expression are genetically modified by inserting heterologous polysaccharide biosynthesis operons into the *wbbL* gene while retaining lipid A-core synthesis. The foreign glycan is built on the inner membrane’s cytoplasmic side using the native Und-PP carrier, flipped to the periplasm by the endogenous Wzx flippase, and then attached to the lipid A-core by the WaaL ligase. Alternatively, engineered glycans can be assembled directly onto the truncated lipid A-core and flipped via MsbA [113]. These modified LPS molecules are transported to the outer membrane and incorporated into vesicles. Because *E. coli* can accommodate diverse plasmid-based glycan biosynthesis pathways, this system serves as a versatile ‘plug-and-play’ platform for displaying glycotopes from various pathogens on OMVs. For example, the gene cluster responsible for the *S. pneumoniae* capsule (Sp-CPS) was introduced into *E. coli* for expression. The Sp-CPS was linked to the lipid A core and was displayed on the surface of both the bacterial cells and geOMVs. Immunizing mice with these geOMVs triggered the production of specific IgG antibodies targeting Sp-CPS and showed effectiveness in opsonophagocytosis studies [204]. In another study, geOMVs were used to develop of a vaccine for uropathogenic *E. coli* (UPEC) serotype, O25b [303]. Chen et al. developed a series of glycoengineered OMVs (glyOMVs) displaying O-polysaccharides from eight different pathogenic bacterial strains, including the highly virulent *Francisella tularensis* subsp. *tularensis* type A strain Schu S4, resulting in ft-glyOMVs. Two weeks after immunization, mice that received ft-glyOMVs showed a two- to three-fold increase in O-polysaccharide-specific IgG levels compared to those immunized with native ftLPS. Additionally, the ft-glyOMVs provided protection against a lethal *F. tularensis* challenge [199].

Notably, host cells can be modified to produce glycosyltransferases and enzymes necessary for the biosynthesis of nucleotide-activated sugars from a range of organisms, allowing for the development of specialized geOMVs [306]. Stevenson et al. developed broadly protective vaccination candidates by synchronizing recombinant poly-N-acetyl-d-glucosamine (rPNAG) production with OMV generation in *E. coli*. The modified bacteria generated glycosylated OMVs that were decorated with the PNAG glycopolymer from *S. aureus*, which induced high levels of PNAG-specific IgG antibodies after immunization in mice. By introducing a *S. aureus* enzyme that facilitates PNAG deacetylation into these cells, geOMVs were produced that triggered antibody responses against both the highly acetylated PNAG and a chemically deacetylated derivative of PNAG. The antibodies generated effectively cleared two distinct PNAG-positive bacterial species: *S. aureus* and the *Francisella tularensis* subspecies *holarctica*. Together, these findings highlight the potential of geOMVs to target conserved polysaccharide antigens and promote immunity against a broad range of pathogens that express surface PNAG [307]. The O-polysaccharide antigen of *S. flexneri* 2a was successfully biosynthesized in *Salmonella* and linked to core-lipid A via the WaaL ligase. Animal studies showed that immunizing mice with the OMV-based vaccine induced a strong specific anti-*Shigella* LPS antibody response in the serum, with similar responses observed in IgA levels from vaginal secretions and bronchopulmonary lavage fluid, following both intranasal and intraperitoneal administration [308].

In conclusion, both glycoconjugated and geOMVs provide an economical and efficient method for evaluating vaccine candidates without using adjuvants in the vaccination process. An added advantage of geOMVs, useful for both vaccines and passive immunization, is their capacity to present a combination of glycans and proteins derived from a specific pathogen. This flexibility could enhance the ability to target a broader range of strains or species with the same geOMVs [303]. However, the function of membrane proteins, including porins, flagellins, and pilins, in eliciting an effective geOMV-mediated immune response must be evaluated.

### 5.8. Nanoparticle-Facilitated OMV Vaccines

Following administration, the transport of vaccines from the site of injection to the draining lymph nodes, along with the dendritic cell maturation and antigen presentation, is crucial for generating a strong antigen-specific immune response. The dimensions of vaccines partially dictate the route of this trafficking. Particulates measuring between 20 and 100 nm primarily migrate to lymph nodes via lymphatic drainage and are more likely to be engulfed by antigen-presenting cells and subsequently delivered to the lymph nodes [309]. The size, shape, and rigidity of particles have been found to impact their uptake by cells, effectiveness in antigen presentation, and activation of APCs [310]. Therefore, techniques for effectively regulating the characteristics of vaccine particles can enhance the immunization efficacy of vaccines. A study by Jang et al. reported that the in vivo kinetic biodistribution of OMV concentration peaked at 3 h post-administration, subsequently declining in most organs within 24 h post-injection [311]. Therefore, the stability of OMVs during in vivo administration needs to be optimized. Gao et al. found out that gold nanoparticles (AuNPs) loaded with OMVs significantly enhanced the OMV stability, leading to improved immunological activation compared to the administration of OMVs alone [200]. Their study demonstrated that AuNPs integrated into OMVs significantly improved the stability of AuNPs in biological buffers, whereas the AuNP core stabilizes the OMVs. Due to their enhanced stability, AuNPs exhibited superior efficiency in stimulating T-cell and B-cell activation by generating elevated cytokine levels and IgG titers [200]. Future research should concentrate on enhancing the stability of OMVs using membrane-coating strategies to improve their efficacy in vaccine development. Synthetic nanoparticles offer highly tunable properties, allowing for precise control over their physical and chemical characteristics. When combined with OMVs, nanoparticle-enhanced OMV vaccines harness the advantages of both platforms, resulting in stronger antigen-specific immune responses. A recent study reported the development of BM-AuNPs by mechanically coating AuNPs with OMVs through multiple extrusion cycles. This process produced membrane-coated AuNPs approximately 30 nm in size. The AuNP core acted as a structural template that transformed polydispersed OMVs into uniformly sized particles and significantly enhanced their stability in biological buffers, likely due to a strong association between the membrane and the core [200]. Following subcutaneous immunization in mice, BM-AuNPs elicited a more robust antigen-specific immune response than unmodified OMVs, as evidenced by enhanced dendritic cell maturation, stronger pathogen-specific antibody production, and improved cellular immune responses. These BM-AuNPs induced higher levels of IFN-γ, IL-17, and more specific antibody responses than OMVs alone. All groups produced low levels of IL-4, suggesting a robust Th1- and Th17-biassed cellular responses [200]. OMVs derived from Carbapenem-Resistant Hypervirulent *Klebsiella pneumoniae* (CRKP) are considered potential candidates for vaccine development. Nonetheless, the immune response to these OMVs remains poorly understood due to challenges in maintaining structural stability and achieving uniform particle size. To overcome these limitations, hollow OMVs from CRKP were reinforced internally with bovine serum albumin (BSA) nanoparticles of controlled size through hydrophobic interactions, leading to a stable and uniform vaccine formulation. The resulting BSA-OMV nanoparticles (BN-OMVs) were approximately 100 nm in diameter and exhibited a well-defined core–shell structure. Administering BN-OMVs via subcutaneous injection led to a substantial rise in CRKP-specific antibody levels. Mice immunized with BN-OMVs exhibited significantly improved survival rates when challenged with a lethal CRKP infection. Findings from adoptive transfer experiments revealed that the protective efficacy of BN-OMVs was driven by both humoral and cellular immune responses [157]. Bong and colleagues reported a novel method for encapsulating AuNPs within OMVs using a bio-orthogonal click reaction for covalent coupling. To achieve this, the outer membrane protein OmpA, which is naturally abundant in OMVs, was modified by incorporating the unnatural amino acid *p*-azidophenylalanine. This introduced azide functionality, allowing for selective covalent binding to alkyne-modified nanoparticles while minimizing effects on healthy tissues [312].

Taken together, integrating well-suited nanoparticle cores has the potential to greatly enhance the potency of OMV-based vaccines by improving antigen stability and immune activation. The choice between protein-based (e.g., BSA) and inorganic (e.g., AuNP) cores depends on the intended application, required immune profile, and production feasibility.

### 5.9. Hybrid OMV-Based Vesicles for Vaccine Development

Membrane fusion is a versatile method that combines two or more cell membrane sources to create a hybrid membrane with advantageous characteristics derived from the original cells, offering a new approach to enhance cell membrane activities. This integration allows for improved functionality by leveraging the distinct advantages of each membrane component [313].

Primary tumour antigens obtained from tumour membranes are shown to elicit both innate and adaptive immune responses that specifically target tumours [314]. The innovative application of membranes derived from cancer cells for various therapeutic purposes has gained significant interest in recent years due to their promising benefits. These membranes, sourced from either patient-derived cancer cells or established cancer cell lines, offer potential solutions to key challenges in cancer treatment, such as personalized therapy, overcoming drug resistance, and enhancing targeted drug delivery. Recently, the fusion of OMVs with tumour cell membranes to create a hybrid membrane for tumour immunotherapy has emerged as a prominent area of interest. Bacterial OMVs and cellular membranes both consist of lipid bilayers, which form the basis of hybrid membrane technology [315]. Diverse techniques, including elevated temperatures, chemical approaches, and electric fields, can be utilized to combine the bacterial membrane with the lipid bilayer of the cell membrane [257]. Moreover, bacterial OMV-based tumour vaccines can be customized for certain tumour types using fusion technology.

OMVs have been combined with various membranes to create hybrid-OMVs, as outlined in the following discussion.

#### 5.9.1. OMV Fusion with Tumour Cell Membrane

To address the limitation of using only a single antigen, OMVs have been merged with tumour cell membranes, which carry a variety of antigens. This combination results in hybrid nanovesicles that include both adjuvants and antigens, promoting a strong immune response [316,317]. Tumour excision yields tumour membrane [318]. OMVs are combined with tumour membrane, sonicated, and then extruded to make hybrid membrane biomaterials [122]. The resulting hybrid bacterial membrane exhibits high stability and can be used to construct drug delivery systems and serve as a carrier for tumour vaccines. Wang et al. employed a hybrid biomimetic membrane (OMV-CC) consisting of bacterial OMVs and melanoma cell membranes to enclose hollow poly-dopamine nanoparticles (HPDA) [319]. Upon intravenous injection through the tail vein in mice, HPDA@[OMV-CC] nanoparticles uniformly targeted melanoma and elicited immune responses by swiftly promoting dendritic cell maturation in the lymph nodes of vaccinated mice. The integration of OMV-CC facilitated targeted drug administration in vivo, hence augmenting the anti-tumour efficacy of nanoparticles [319]. Li et al. combined OMVs with tumour cell membranes to develop vesicles named BTs to create a BTs nano-vaccine. This vaccine efficiently activated the immune system, facilitated dendritic cell activation, induced TLR and cGAS-STING signalling pathways, and augmented the lethal effects of particular cytotoxic T lymphocytes on tumour cells. During in vivo tests, administration of the BTs nano-vaccine inhibited the proliferation of B16-F10 tumours and lowered their fatal spread [317]. In a separate investigation, melanoma cytomembrane vesicles (CMVs) were combined with *Salmonella* OMVs to enhance functionality and expandability for improved immunization outcomes [316]. Animal studies on the preventive application of this vaccine indicated that EPV functions as a protective vaccine by stimulating the immune system and triggering an antitumour response, thereby inhibiting cancer development [316]. Zou et al. developed a hybrid vesicle system to augment the innate immune responses and promote tailored immunotherapy. The tumour-derived cell membrane (mT) was integrated with OMVs to develop specialized vesicles, termed mTOMV. It was revealed by in vitro studies that mTOMV effectively stimulated innate immune cells and strengthened T-cell-mediated tumour lysis. Furthermore, mTOMV accumulated in the inguinal lymph nodes, leading to a significant reduction in lung metastases. It also triggered an adaptive immune response specifically against homologous tumours, underscoring its potential for personalized immunotherapy. The ability of mTOMV to prevent tumour development and metastasis, along with excellent biocompatibility and a straightforward production process, underscores its significant potential for therapeutic applications [201]. In another study, a heterogenic membrane-based biomimetic hybrid nanoplatform (MGTe) was developed by merging glutathione (GSH)-decorated tellurium (Te) nanoparticles with fused tumour cell membranes and OMVs [320]. In a recent study, breast cancer cell membranes were fused with OMVs to create a hybrid membrane (HM), which was then used to coat IR780-loaded poly (lactic-co-glycolic acid) (PLGA) nanoparticles. The resulting formulation, IR780@PLGA@HM, exhibited tumour-targeting properties, immune-modulating effects, and sonodynamic activity [321].

In summary, immunotherapy alone frequently fails to entirely eradicate solid tumours [322]. Therefore, combining immunotherapy with other therapeutic modalities, such as a hybrid OMV-based tumour vaccine, could be an effective therapeutic strategy.

#### 5.9.2. Bacteria–Plant Hybrid Vesicles (BPNs)

The plant-derived thylakoid membrane is a distinct type of membrane encompassing photosystems and numerous enzymes capable of inducing efficient photodynamic effects [323]. These membranes are becoming a formidable possibility for inducing ICD, which can directly eliminate tumours and simultaneously release whole-cell tumour antigens, particularly neoantigens generated by mutations during tumour growth [324].

In a recent study, bacteria-plant hybrid vesicles (BPNs) were created by merging the thylakoid membrane with OMVs. Following intravenous administration in mice, BPNs preferentially concentrated in tumour tissues and stimulated the dendritic cell maturation owing to the exceptional targeting capacity and adjuvant properties of OMVs [325]. Upon laser irradiation, BPNs produced a significant quantity of reactive oxygen species (ROS), inducing effective ICD and the consequent secretion of tumour-associated antigens (TAAs) and damage-associated molecular patterns (DAMPs). Additionally, BPNs have the ability to reprogram M2 macrophages into the M1 phenotype, which enhances the photodynamic effects and stimulates immune responses by mitigating the immunosuppressive tumour microenvironment. This capability helps improve immune responses within the tumour environment [325]. Consequently, both tumourigenesis and metastasis can be effectively inhibited. Thus, BNPs present a promising approach for developing a multifunctional membrane-based hybrid system for effective therapies.

#### 5.9.3. Lipid Hybrid OMVs

Liposomes are synthetic bilayer membrane structures characterized by a significant drug-loading potential and ease of preparation and modification. Although these materials can address some limitations of cell-derived biomimetic functional materials, they naturally lack inherent active targeting capability. To enhance their functionality, lipids can be combined with cell membranes, EVs, or OMVs, forming lipid hybrid biomimetic materials. This hybrid approach overcomes the disadvantages associated with both cell-derived elements and liposomes, making them a promising alternative for vaccine development [30]. This hybridization also lowers the occurrence of acute systemic immune responses. Researchers have engineered bacterial-derived biomimetic functional materials by incorporating thermosensitive liposomes loaded with the CD38 siRNA and photosensitizer Cypate. These nanocarriers demonstrated tumour-targeting capabilities, accumulating at the tumour site and inducing cancer cell destruction through laser-triggered thermal ablation. Photothermal therapy enhanced antigen exposure by initiating ICD, thereby facilitating antigen presentation and stimulating antitumour immune responses. Furthermore, the inhibition of CD38 improved T-cell function within the tumour microenvironment, reinforcing immune activity against cancer [30]. Another hybrid system was developed by combining cationic liposomes, a PD-L1 trap plasmid, tumour cell-derived exosomes, and *Akkermansia muciniphila*-OMVs (Akk-OMVs). This formulation, known as Lipo-PD-L1@HEV, effectively reached the lymph nodes, promoted dendritic cell maturation and cytotoxic T lymphocyte activation, thus presenting a promising approach for gene-therapy-driven cancer vaccination and synergistic immunotherapy [321].

In summary, the integration of bacterial OMVs with other cell-derived components offers a versatile and effective strategy for vaccine development and drug delivery, paving the way for the development of next-generation therapeutics.

### 5.10. In Situ Production of OMVs by Genetically Engineered Bacteria

**Figure 5:**
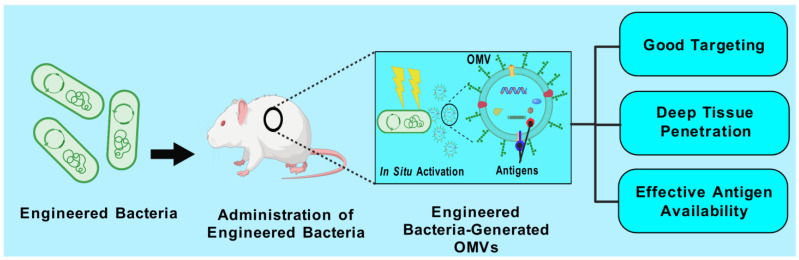
In situ release of engineered outer-membrane vesicles (OMVs) from transplanted bacteria. Genetically modified bacteria can be administered to a host to actively generate and release OMVs in situ. These OMVs subsequently deliver their surface-associated and internal biomolecules into the membranes or cytoplasm of target cells, facilitating precise biological functions.

Despite the increasing number of technologies for isolating and purifying vesicles, none adequately fulfil therapeutic requirements [326]. Existing isolation techniques, including ultra-centrifugation, size exclusion, immunoaffinity, and microfluidics, do not ensure the efficient production and/or purity of OMVs [327]. These isolation processes typically encompass numerous steps, rendering them arduous. Moreover, the separated OMVs typically necessitate additional functional modification or drug loading. Methods like sonication, extrusion, and the use of organic solvents are frequently utilized in the cargo-loading procedure, potentially compromising the integrity and functionality of biological vesicles [328]. The functions of cargo including proteins and internal nucleic acids, along with the integrity of biological vesicles, are essential to the therapeutic efficacy of OMVs. Consequently, employing OMVs as therapeutic agents continues to encounter challenges. Therefore, novel techniques must be established to prevent unproductive efforts during the functionalization and in vitro separation of OMVs. Recent findings reveal that bacteria and cells have the intrinsic ability to generate biological vesicles within the body, which can serve functional and therapeutic roles. In 1975, the researchers DeVoe and Gilchrist discovered that meningococcus-infected patients could release OMVs into their cerebrospinal fluid [8]. Further studies have demonstrated that bacteria naturally produce OMVs within infected organs and the gastrointestinal tract [329]. Therefore, employing organisms to generate endogenous biological vesicles within the body to transport bio-information and deliver drugs has emerged as a promising therapeutic strategy. Recent reports indicate that external stimulus can modify the production and functionality of endogenous biological vesicles, imparting therapeutic qualities to them [330]. Thus, to tackle the complex production method for exogenous biological vesicles, a unique technique has been developed: employing bacteria for the endogenous release of biological vesicles as vehicles for vaccine and bio-information transport, as shown in Figure 5. Unlike conventional biological vesicles that naturally exist in vivo, these endogenous vesicles are modified, activated, or functionalized by external materials within the body before being released at the site of action. To confer therapeutic activities by the biological vesicles generated by bacteria, these bacteria are first functionalized. Subsequently, functional OMVs are discharged into the body. The transplantation of organisms for the active release of endogenous vesicles into the body can prevent the loss associated with extracting vesicles in vitro.

Yue and colleagues reported that genetically modified *E. coli* can produce OMVs displaying a specific tumour antigen fused to the ClyA protein in situ. This expression is regulated by a promoter responsive to the presence of the monosaccharide arabinose. In murine models, the ingestion of arabinose along with genetically modified *E. coli* triggered the generation of OMVs, which traversed the intestinal epithelium and reached the lamina propria, where they facilitated dendritic cell maturation [208]. OMVs carrying the antigen successfully restricted tumour growth in mice with lung metastatic melanoma and subcutaneous colon tumours, while also protecting against subsequent tumour re-challenge. In comparison with the altered bacteria devoid of Ara (ClyA-OVA-mFc(-Ara)), the engineered bacteria having Ara (ClyA-OVA-mFc) demonstrated a markedly reduced incidence of lung metastases, indicating the critical function of the responsive switch [208]. Another investigation reported that the oral delivery of OMVs derived from modified *E. coli* Nissle and *E. coli* BL21(DE3)ΔompA, harbouring cancer-specific T-cell epitopes, induced epitope-specific T-cell responses in the gut, and suppressed tumour growth in mice. Shotgun sequencing of the microbiome and T-cell receptor sequencing from T-cells obtained from both tumours and lamina propria demonstrated that the primary process of tumour suppression is facilitated by the activation of cross-reacting T-cells at the intestinal site, which then migrates to the tumour microenvironment [331].

In essence, these approaches have the potential to overcome both the challenges of commensal bacteria penetrating the epithelial barrier and the complexities of the digestive tract that hinder conventional oral vaccines. Genetically engineered commensal bacteria that generate OMVs in situ to deliver stimulatory substances could contribute significantly to the development of oral vaccines and therapeutics. The production of OMVs in certain regions of the body can be achieved through particular bacterial alterations. OMVs produced this way may yield better therapeutic outcomes, particularly for deep tumour penetration. However, the technique of genetically modifying organisms, introducing them into the host, and subsequently producing OMVs in situ necessitates enhanced biosafety measures throughout the procedure. Furthermore, the cessation of in situ vesicle production post-treatment is an issue that warrants consideration. It can be anticipated that these constraints will be progressively surmounted with the advancement of new technology, and this innovative technique will be extensively utilized across various diseases and facilitate the convergence of multiple fields.

While OMVs offer significant immunostimulatory potential, practical aspects, such as delivery routes, in vivo stability, and formulation strategies, must be carefully considered. OMVs can be administered through multiple routes, including oral, intranasal, intramuscular, subcutaneous, intraperitoneal, and intradermal methods depending upon the targeted pathogen and desired immune response [113]. While intramuscular delivery is effective for inducing systemic immunity via local lymph node activation, mucosal routes, particularly intranasal ones, are more effective for respiratory pathogens as they stimulate both systemic and mucosal immune responses [196,332]. Nonetheless, intranasal formulations face challenges, such as rapid mucociliary clearance and the need for mucoadhesive delivery systems to enhance retention time and absorption. In terms of in vivo stability, OMVs exhibit relatively good structural resilience under various storage and physiological conditions. Studies have shown that OMVs can retain their integrity and immunogenicity after lyophilization or exposure to physiological temperatures [61,333]. However, concerns remain regarding potential alterations in OMV morphology, aggregation, or antigen degradation following prolonged circulation in biological fluids [200]. These issues are particularly relevant for mucosal or systemic delivery routes, where OMVs may encounter diverse pH levels, immune components, and biochemical environments. While direct evidence of enzymatic degradation in vivo remains limited, maintaining vesicle stability and antigen integrity under physiological conditions is an ongoing challenge that necessitates further formulation refinement, such as embedding OMVs into protective delivery matrices or encapsulating them in nanoparticles, to enhance durability and bioavailability [157]. However, key aspects, such as formulation optimization and delivery-related stability, remain underexplored, highlighting the need for systematic studies to guide rational design and support clinical translation.

## 6. OMVs for Multi-Antigen and Multi-Pathogen Vaccine Development

Vaccines were developed more than 200 years ago, with the recombinant vaccines era commencing in 1981 [334]. Notwithstanding this extensive experience, the development of effective vaccines for new infections continues to be challenging and retains a considerable empirical aspect. From this perspective, the advancement of novel technologies to enhance vaccine design should be considered. The vaccine industry holds particular importance for low- and middle-income nations, which house a significant portion of the global population, where infectious illnesses result in severe health and socio-economic repercussions [64]. It is estimated that infectious diseases account for 91% of fatalities in low- and middle-income nations [335]. For example, Tuberculosis is a disease mostly induced by poverty that exclusively impacts impoverished nations [336]. Similarly, diarrheal diseases continue to be the second leading cause of mortality in children under five, especially in low-income nations where limited access to clean water, adequate sanitation, and proper hygiene remains a significant concern. Although these preventive measures are standard in high-income nations, their implementation is often difficult in resource-limited settings. As a result, vaccination serves as the most effective tool in the global fight against microbial infections, especially in the most disadvantaged regions [337]. The rise in circulating antimicrobial-resistant strains presents a significant risk of diseases becoming untreatable, perhaps leading to a worldwide health disaster [338,339]. Pathogens frequently employ mutation-driven alterations of surface antigens and variable expression of virulence, adhesion, and colonization components to evade the host immune system. These evasion strategies render a pathogen a constantly changing target for the immune system and pose a difficulty for vaccine design, as vaccines must be made with many—and, at times, several—antigens to ensure adequate coverage. To tackle these issues, the development of multi-antigen and multi-pathogen vaccines is a strategic response. For example, the latest Human Papillomavirus and pneumococcal vaccines comprise five variations in the L1 protein and up to 15 glycoconjugates, respectively, whilst the Meningococcus B vaccine and the acellular *B. pertussis* vaccine each contain five unique virulence factors [340]. Multi-antigen vaccines improve the breadth and depth of immune protection by eliciting robust and diverse immune responses, reducing the likelihood of immune escape. Multi-pathogen vaccines, on the other hand, address co-infections that exacerbate disease severity, such as the combined role of NTHi and *Moraxella catarrhalis* in otitis media [341], offering a holistic approach to disease prevention. In addition to enhancing immune responses, multi-antigen vaccines simplify immunization schedules by combining multiple antigens or pathogens into a single formulation, minimizing the number of injections required. Nonetheless, the development of multi-component or multi-pathogen vaccines can be complicated. Therefore, the accessibility of the latest platforms that facilitate such vaccine design is exceedingly advantageous. Recent advancements in vaccine technologies have revolutionized the design and delivery of multi-antigen and multi-pathogen formulations. These innovations allow for the inclusion of diverse, highly conserved, and engineered antigens while maintaining stability, safety, and immunogenicity. Moreover, such vaccines have the potential to reduce costs associated with manufacturing, distribution, and administration, making them more feasible for global immunization programmes.

OMVs represent a promising platform for multi-antigen and multi-pathogen vaccine development due to their structural and immunostimulatory properties. As detailed above, they can be engineered to display antigens from multiple pathogens, addressing co-infections and polymicrobial diseases. OMVs are also easy to produce, and their ability to carry multiple antigens in a single vesicle can streamline multivalent vaccine development. Their intrinsic adjuvant effects reduce the need for additional adjuvants. This versatility supports the development of broad-spectrum vaccines targeting antigenic variability and complex host–pathogen interactions. Recent studies (see Table 4) have demonstrated promising immunogenicity and protection in animal models using engineered OMVs with multiple antigens from the same or different pathogens. It remains to be seen how these findings translate to clinical settings and which candidates advance to human trials.

## 7. Breakthrough of Artificial Systems Mimicking OMV Features

### 7.1. Cellular Nanodiscs Based on OMVs for Vaccine Development

**Figure 6:**
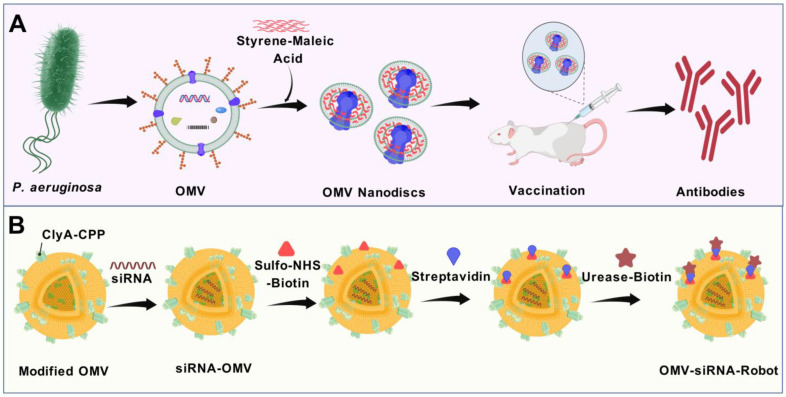
Preparation and engineering of OM-NDs and OMV-based nanorobots. (**A**) Outer-membrane vesicles (OMVs) derived from *Pseudomonas aeruginosa* are isolated and treated with styrene-maleic acid (SMA), resulting in the formation of OM-NDs. This formulation serves as a potential nano-vaccine for protection against bacterial infection. The figure is adapted from [355]. (**B**) Fabrication and characterization of OMV-siRNA robots. The panel depicts the fabrication process of OMV-siRNA nanorobots, featuring surface-engineered CPP designed for targeted tumour binding and penetration. Biocatalytic propulsion improves the targeted binding and penetration of siRNA-loaded OMV nanorobots (OMV-siR robots) at the tumour site. The figure is adapted from [356]. ClyA-CPP: Cytolysin A-fused cell-penetrating peptide; siRNA: Small interfering RNA; OM-NDs: Outer-membrane vesicle-based nanodisc; OMV: outer-membrane vesicle.

Protein-containing lipid aggregates called nanodiscs are made up of 100–200 lipid molecules and are enclosed in amphipathic helical peptides or membrane scaffold proteins, which are usually produced from apolipoprotein A-1 [353]. After being administered subcutaneously, cellular nanodiscs easily move through the lymphatic system, allowing for the transfer of adjuvant and antigen payloads to immune cells found in draining lymph nodes. Therefore, nanodiscs have demonstrated their efficacy as vaccine candidates by strongly promoting adaptive immunity in a way that is specific to antigens [354]. Noh et al. expanded the use of nanodisc technology to natural cell membranes and described the use of cellular nanodiscs made from bacterial OMVs (referred to as OM-NDs) in place of a synthetic lipid bilayer [355]. Styrene-maleic acid (SMA), an amphiphilic membrane scaffold copolymer, was incubated with *P. aeruginosa* OMVs to formulate OM-NDs, as shown in Figure 6A. Compared to conventional OMVs, OM-NDs were found to more efficiently reach the draining lymph nodes following subcutaneous delivery due to their tiny size. Additionally, OM-NDs stimulated the maturation of dendritic cells and produced antibodies specific to the pathogen. When challenged intratracheally with live *P. aeruginosa*, vaccinated mice had increased longevity, and this protection was also linked to a decrease in lung local inflammation. Overall, this study shows that bacterial OMV-derived nanodiscs can be used as vaccines to prevent bacterial infections [355].

Overall, nanodiscs are known to have improved lymphatic trafficking and antigen presentation efficiency. Due to their smaller, uniform size and enhanced biophysical properties, nanodiscs can more effectively drain into lymph nodes, thereby promoting better uptake by dendritic cells and stronger activation of adaptive immunity [357]. Nonetheless, realizing the clinical utility of nanodiscs will require sustained research efforts, thorough validation, and progression through clinical trial phases.

### 7.2. OMV-Based Nanorobots

Nanorobotic technology is becoming a ground-breaking approach for accurate diagnosis and treatment. Much work has gone into creating fascinating nanorobots that are powered by chemical reactions or external forces [358,359]. Steerable nanomachine motion in small areas has demonstrated potential for increasing the effectiveness of cellular diagnosis and treatment, including improved cellular internalization [360,361], efficient drug delivery [362,363], and rapid intracellular sensing [364,365]. However, inorganic materials or metals [366,367], metal–organic frameworks [368], and polymers [369,370], are the primary building blocks of nanorobots, and their biocompatibility and biodegradability are compromised. The inorganic structure of contemporary nanorobots has insufficient inherent biofunctionality, barely satisfying the requirements for practical applications, and lacks the characteristics necessary for precise binding and penetration into biological tissues. In contrast, biological activities, including tumour targeting, tissue penetration, and immunological control, constitute another dimension that are required to improve nanorobot efficacy in executing medical tasks [371,372]. Recently, OMV-based nanorobots have been developed. These nanorobots maintain many intrinsic features and functions of nanoscale OMVs, including enhanced biocompatibility, bacterial macromolecules (such as LPS, proteins, DNA, and peptidoglycan) that trigger immune activation, and the flexibility for genetic bioengineering to generate targeted functional proteins. The stable membrane architecture facilitates drug encapsulation within OMV nanorobots, minimizing leakage and enzymatic degradation [122]. Researchers developed enzyme-driven bacterial OMV nanorobots, where urease immobilized on the OMV membrane breaks down bioavailable urea, providing effective propulsion for the nanorobots. This OMV-based nanorobot preserves key features of OMVs, including natural biocompatibility, immunogenic properties, flexible surface bioengineering for tailored functionalities, and the ability to load and protect cargo. The robotic body was bioengineered with cell-penetrating peptides (CPP) to facilitate targeting and penetration of tumours, augmented by the active propulsion of nanorobots, as shown in Figure 6B. Furthermore, OMV nanorobots could proficiently protect the encapsulated siRNA from enzymatic degradation. By conducting comprehensive in vitro and in vivo experiments using a rodent model, researchers demonstrated that these OMV-based nanorobots markedly enhanced siRNA delivery and immune activation. This improvement resulted in superior tumour suppression, particularly in the orthotopic bladder tumour model, when compared to non-motile control groups [356]. While the OMV-siRNA nanorobots present a highly innovative strategy, their complex architecture, involving multiple biomolecular components, may pose challenges for large-scale production and clinical translation. Nevertheless, their modularity and promising performance in preclinical models suggest potential for future development in biomedical applications, provided that further optimization and simplification are achieved.

## 8. Outlook on OMV Bioengineering Using Synthetic Biology Approaches

Over the past few decades, synthetic biology has emerged as a rapidly evolving, interdisciplinary field focused on deciphering biological complexity in a structured manner. Its goal is to strategically engineer biological systems for innovative and practical applications [373]. As a result, this field has cultivated a diverse set of sophisticated techniques and technologies, guided by an engineering-driven approach and ethical innovation. These advancements have significantly accelerated the translation of research into practical, real-world applications [374]. The integration of synthetic biology offers a transformative approach for advancing OMV research and accelerating its applications across multiple fields. This section examines how the growing integration of OMV research with cutting-edge synthetic biology methods can foster groundbreaking advancements. This collaboration has the potential to revolutionize the development of novel therapeutic solutions, enhancing the precision and efficacy of treatments.

**Figure 7:**
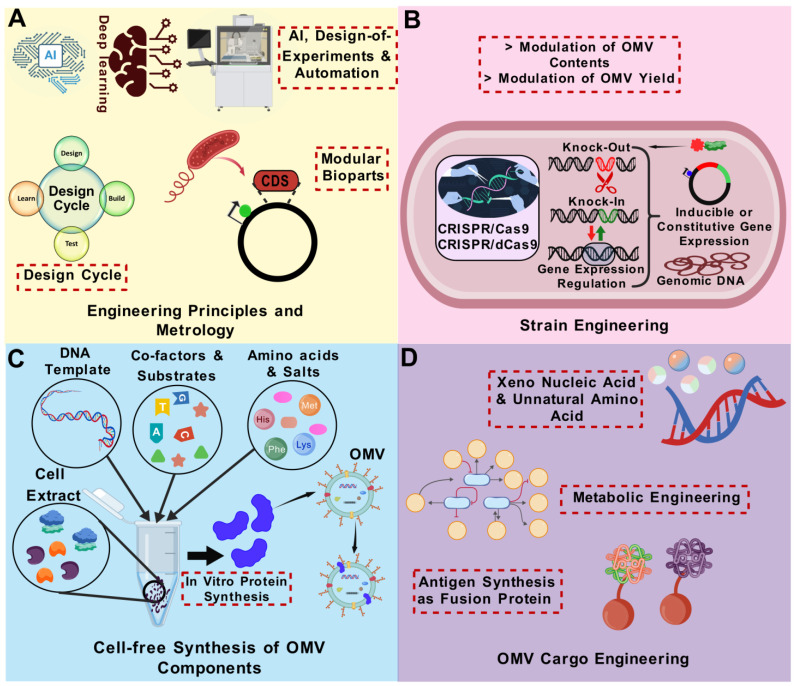
Synthetic biology strategies for engineering outer-membrane vesicles (OMVs). (**A**) Potential synthetic biology-based engineering strategies that can be applied to modify OMV-producing strains. These modifications aim to enhance OMV production and optimize the therapeutic cargo of microbially derived OMVs. (**B**) Schematic representation of the cell-free synthesis of OMV components, utilizing cell-free protein synthesis for OMV assembly. (**C**) Overview of strain-engineering approaches for OMV production, including CRISPR/Cas9-based gene regulation, knockout and knock-in strategies, and inducible gene expression to modulate OMV yield and content. (**D**) For OMV cargo engineering, xeno nucleic acids, unnatural amino acids, and metabolic engineering can be incorporated to generate antigens for therapeutic and vaccine applications. AI, artificial intelligence; CDS: coding sequence; CRISPR/dCas9: clustered regularly interspaced short palindromic repeats (CRISPR)/endonuclease deficient CRISPR-associated protein 9 (dCas9); OMV: outer-membrane vesicle.

In the context of OMV engineering, bacterial strains with modified OMV payloads could be systematically engineered using a design–build–test–learn (DBTL) cycle method (Figure 7A). Zanella et al. demonstrated this approach by utilizing CRISPR/Cas9 genome editing to precisely delete 59 native OMV-associated protein genes in a genetically modified *E. coli* BL21(DE3)Δ60 strain [375]. This research provided a valuable understanding of how OMV protein cargo is naturally integrated within *E. coli* BL21. Additionally, it demonstrated a genetic engineering approach to expand the incorporation of foreign antigens into OMVs.

Synthetic biology is also advancing genome editing tools, such as zinc-finger nucleases (ZFNs), transcription activator-like effector nucleases (TALENs) [376], and nucleobase deaminase enzymes, as well as gene expression regulation approaches like catalytically dead CRISPR/dCas9 [377], which can be utilized in future OMV engineering research. An essential application could involve employing advanced strain engineering techniques to precisely adjust bacterial/OMV LPS levels, optimize the display of polysaccharide antigens on OMV surface, or modify the concentration of other immunomodulatory substances to reduce undesirable toxicity (Figure 7B). To increase strain coverage, OMVs from various strains could be combined to enhance vaccine efficacy [300,301]. Advancements in synthetic biology have enhanced the range of gene regulatory elements, such as promoters, along with functional genetic components like periplasmic localization tags [378,379]. Their modular design and seamless integration with cutting-edge DNA assembly methods have further enhanced their applicability [373,380]. Moreover, cell-free protein synthesis (CFPS) systems, which leverage isolated cellular transcription and translation mechanisms, offer a platform for testing and optimizing different expression plasmids or cargo configurations (Figure 7C). This approach can accelerate the iterative process of OMV engineering and design [374,381]. Recent advancements in protein modelling and folding, such as AlphaFold [349], large language models for proteins (e.g., ESM-2) [382], and advanced computational tools [383,384], which have significantly enhanced the ability to design and predict protein structures and functions with greater accuracy, may be utilized in forthcoming OMV research to engineer completely novel OMV cargo proteins.

Advances in bacterial metabolic engineering, such as non-natural amino acid incorporation or codon reassignment [385], and xeno nucleic acids (XNAs) [386], could lead to the creation of highly effective OMV cargoes and medical interventions that are synthetic and independent of the biochemistry of the host cells (Figure 7D). The fusion of synthetic biology with advanced OMV engineering techniques holds the potential to facilitate the generation of synthetic membrane vesicles that are derived from cells that have been entirely engineered. This innovative approach could open up new possibilities for designing customized vesicles with precise functions, independent of natural cellular processes, and could pave the way for groundbreaking applications in medicine and biotechnology.

The integration of omics technologies and artificial intelligence (AI) can also transform OMV engineering. Proteomic and lipidomic profiling of OMVs enables comprehensive mapping of cargo content, informing targeted modifications for improved safety and antigen presentation [375,387]. Transcriptomics also helps elucidate the regulatory pathways influenced by OMVs [388]. The integration of machine learning algorithms can open new avenues for predicting vesicle yield, cargo loading efficiency, and immunostimulatory potential across different bacterial strains [389]. Although these approaches are still in the early phases for OMV vaccines, they promise to increase precision, scalability, and safety in next-generation vaccine development.

### 8.1. Synthetic OMVs

To simplify the inherent complexity of OMVs for the examination of specific components, Kehl et al. devised a methodology to generate an OMV-like environment devoid of LPS and/or flagellin, facilitating the analysis of related virulence factors in controlled conditions. To do this, they modified established techniques for liposome/vesicle preparation by employing a unique lipid composition, and they subsequently incorporated the appropriate proteins, resulting in products referred to as synthetic OMVs (sOMVs). Following the establishment of several metrics utilizing bovine serum albumin (BSA) as a proxy, the authors successfully generated sOMVs encapsulated with Stx2a, the Stx subtype of Shiga toxin (Stx). The study presents a method for examining individual virulence factors, particularly toxins, within an OMV-like context [390]. In another study, nitrogen cavitation was used to develop double-layered membrane vesicles (DMVs) from *P. aeruginosa.* Cryo-transmission electron microscopy (Cryo-TEM) and proteomics analyses demonstrated that DMVs maintained the integrity of the bacterial membrane and included a broad array of membrane proteins essential for immunogenicity. Immunization with DMVs increased survival rates in *P. aeruginosa*-induced mouse sepsis model [391]. This novel and simple technique utilizing nitrogen cavitation holds great potential for advancing vaccine development.

In another approach, researchers developed synthetic bacterial vesicles as an alternative to OMVs. They fragmented bacteria debris using lysozyme-induced lysis followed by sonication. To remove the inner bacterial membrane, sarkosyl (an ionic detergent) was employed, and high-pH conditions were applied to remove cytoplasmic components from the debris. This approach produced a good yield, characterized by low cellular elements and a complete lack of RNA or DNA [392].

Taken together, synthetic OMVs have the potential to revolutionize vaccine development by offering a controlled environment to study and target specific virulence factors, such as toxins, in a precise manner.

### 8.2. A Step Towards Scalable OMV Vaccine Technology: Emerging OMV Isolation and Production Methods

Technical methods for vesicle assembly currently lack definitive standards, and large-scale manufacture of OMVs remains unfeasible. The regulation of OMV size is challenging, with considerable variability observed among batches. Furthermore, limited particle programmability results from an insufficient understanding of the exact OMV assembly mechanism. Improving safety and minimizing expenses remain issues that require attention. In contrast to EVs obtained from animal cells, which face limitations like limited yield and challenges in industrial-scale manufacture, OMVs may be favourable to mass production. This is primarily due to bacteria’s capacity for fast reproduction and their ability to be cultivated at large densities. At present, ultracentrifugation and density gradient centrifugation are the predominant methods employed for the separation and purification of OMVs [393]. Nonetheless, these techniques are laborious and time-consuming, necessitating the acquisition of costly ultracentrifuge equipment. Consequently, there is a need for rapid, efficient, and convenient techniques for the separation of OMVs. A recent study established a novel economical gradient filtering technique for the separation of OMVs, capable of industrial-scale production while preserving the physiological functions of the isolated OMVs. By utilizing filter membranes with varying pore sizes (300 nm and 100 kDa), a system was established that allowed for small molecular substances to pass through while retaining larger particles, effectively enabling separation [394]. This research evaluated the gradient filtration technique against conventional ultracentrifugation for isolation of *E. coli* Nissle 1917 (EcN) OMVs. Subsequently, the study employed RAW264.7 macrophages as an in vitro model to study the impact of EcN-derived OMVs procured via the gradient-filtering approach on immunological function. The results demonstrated that OMVs originating from EcN were effectively separated using the new gradient filtration technique. The OMV enrichment level attained with this gradient filtering method was almost twice as effective as that obtained by conventional ultracentrifugation [394]. This innovative and straightforward separation technique may possess significant potential for applications related to the evaluation of OMVs. Another study investigated an alternate purification method that integrates size-exclusion chromatography (SEC) using zeolite columns. SEC is recognized for its capacity to produce substantial amounts of mammalian EVs while preserving their shape [395]. This strategy enabled the separation of vesicles of around 200 nm from smaller, unwanted molecules of around 50 nm. Despite zeolite’s primary drawback of releasing small particles that could contaminate the collected fractions, it possesses the potential to serve as a dependable, economical, and swift approach for purifying OMVs [396]. A high-yield magnetic extraction method for *E. coli* OMVs was reported, in which *E. coli* was grown with magnetic iron-oxide nanoparticles (MNPs). The proliferation of *E. coli* in the presence of PEGylated MNPs increased OMV secretion and OMV gene upregulation. MNP-containing OMVs can be magnetically recovered 60 times more efficiently than ultracentrifugation. Bacteria absorbed both magnetically harvested and ultracentrifugation-derived OMVs in similar amounts. Furthermore, such magnetic OMVs can be directed to specific delivery sites in the human body through the application of magnetic fields [397]. Another study developed a dual differential gradient centrifugation (DDGC) approach for *K. pneumoniae* OMVs by combining differential gradient centrifugation (DC) with one-step ultracentrifugation (ODG). The study showed that DDGC-isolated OMVs had a standard morphology, less contamination, and greater homogeneous particle size distribution compared to DC-extracted OMVs [398]. Moreover, the hydrostatic filtration dialysis (HFD) method has been modified and utilized for the extraction of OMVs in many reported studies [399,400]. This approach has proven to be a cost-effective, straightforward, and dependable method for isolating OMVs, particularly in experiments necessitating substantial quantities of OMVs, such as in vivo immunization studies. A further benefit of HFD is its remarkable consistency in reproducibility between batches, a trait essential for vaccine production [274]. Current data regarding the use of HFD to isolate OMVs is insufficient to provide a thorough comparison with other previously described OMV isolation strategies. Nevertheless, this approach holds great potential for the large-scale manufacturing of OMV-based vaccines.

Current OMV production procedures are batch-based. Batch-produced OMVs have poor yields along with substantial costs. High-yield OMV production may be possible with continuous bioprocessing to increase equipment utilization, shorter cycle durations, and lower facility footprints [401]. This reduces production and investment expenses. OMV production methods could be switched from batch to continuous to reduce production costs, boost volumetric productivity, and improve product quality. Notably, the FDA also promotes continuous biopharmaceutical production [402]. In *N. meningitidis*, moving from batch to continuous OMV synthesis enhanced production nine-fold at a given dilution rate. A replicable steady state could be maintained for 600 h. There was no change in OMV properties during the testing, proving that a continuous production method for any application is possible [120]. Changing the production process enhanced productivity without impacting OMV quality. A similar method may boost OMV yields in other bacteria. Additionally, reversible phase-separation approaches, such as those using elastin-like polypeptides [403,404], could be used for faster and easier recovery.

Despite the challenges in standardizing vesicle assembly and optimizing large-scale OMV production, recent advancements in purification and production techniques offer promising solutions. As these techniques evolve, they could make OMV-based vaccine production more feasible on an industrial scale, enhancing their potential for global health applications.

## 9. Regulatory, Manufacturing, and Translational Challenges in OMV-Based Vaccine Development

The translation of OMV-based vaccines from the laboratory to clinical use presents a complex set of challenges that span regulatory approval, manufacturing scalability, and clinical development. While OMVs offer promising immunogenic properties and unique engineering flexibility, the path to safe, effective, and marketable vaccines is not without hurdles. One of the primary regulatory challenges is ensuring the safety and immunogenicity of OMVs in humans. Preclinical studies must demonstrate not only efficacy, but also an acceptable safety profile, particularly given the presence of endotoxins such as LPS, which are intrinsic to OMVs and can trigger unwanted inflammatory responses. Genetic detoxification strategies or structural modifications are often employed to mitigate this risk, but each adaptation introduces additional layers of regulatory scrutiny. In the United States, the FDA requires submission of a comprehensive Investigational New Drug (IND) application before initiating clinical trials. This application must include robust data on preclinical testing, quality control procedures, and detailed manufacturing protocols [405]. Similarly, in Europe, the European Medicines Agency (EMA) mandates a full dossier of non-clinical and clinical data, safety assessments, and manufacturing details [406]. However, a major gap in both regions is the lack of clear OMV-specific regulatory frameworks. Most developers must interpret general biologics or vaccine guidelines and adapt them to the unique structural and biological characteristics of OMVs. This reliance on generalized pathways can delay progress and introduce uncertainty in the development process. Adding to this complexity is the issue of batch-to-batch consistency. OMVs are biologically derived and influenced by bacterial strain variations, cultivation conditions, and purification protocols [120]. These factors can lead to heterogeneity in size, protein content, and immunostimulatory properties, making standardization difficult. Therefore, establishing robust manufacturing pipelines with reproducible and tightly controlled parameters is critical. This includes developing advanced characterization tools for OMV profiling and ensuring uniformity across production lots.

Good Manufacturing Practice (GMP) compliance forms the backbone of clinical-grade OMV production. However, current OMV production techniques, such as ultracentrifugation, are labour-intensive, low-yield, and challenging to scale [407]. To overcome these limitations, emerging alternatives like tangential flow filtration and size-exclusion chromatography are being explored for their scalability and GMP compatibility [408]. In parallel, continuous manufacturing platforms already endorsed by regulatory agencies like the FDA show promise for improving yield, reducing production costs, and maintaining consistent quality across extended manufacturing runs. As discussed earlier, continuous bioreactor systems for *N. meningitidis* have demonstrated stable, high-yield OMV production [120]. Facility design is another essential factor in meeting GMP standards. Infrastructure must support sterile processing environments, biocontainment for pathogenic strains, and rigorous cleaning and documentation procedures. This includes the use of HEPA-filtered cleanrooms, automated sterilization systems, and biosafety cabinets to ensure product safety and operator protection.

Beyond manufacturing and regulation, translational hurdles persist in clinical development. One notable challenge is the lack of universally accepted correlates of protection for OMV-based vaccines. This complicates trial design and efficacy assessment, often necessitating larger and more complex clinical studies. Moreover, inter-individual variation in immune responses driven by genetics, age, or geographic exposure can further affect vaccine performance, underscoring the importance of diverse clinical trial cohorts [409]. Lastly, navigating intellectual property laws and obtaining regulatory approvals across multiple jurisdictions can delay global deployment. Strategic alignment with regulatory agencies, early planning for global market entry, and open dialogue about OMV-specific risks and benefits are essential to streamline clinical translation.

In summary, while OMVs offer transformative potential as next-generation vaccine platforms, their successful clinical development demands coordinated advances in regulatory clarity, scalable GMP manufacturing, and rigorous translational research. Addressing these challenges through cross-disciplinary collaboration will be critical to realizing the full promise of OMV-based vaccines.

## 10. Concluding Remarks

The unexploited potential of modified OMVs heralds a new era of opportunity in the evolving field of immunotherapy, promising to transform our approach to combating difficult diseases. After discovering bacterial OMVs about 50 years ago, scientists have continued to study their mechanisms and impacts. OMVs remain somewhat ambiguous, like exosomes and eukaryotic EVs. The fact that we have yet to discover their biogenesis mechanism(s) after 50 years of inquiry raises many concerns. Various species have revealed some aspects of OMV biogenesis, but none of the functioning models are universal. Cutting-edge microscopy and mass spectrometry techniques may equip researchers with the ability to explore the vesiculation process in clinical strains and infection models, shedding light on OMV formation and its functional significance. Several questions remain: How and why are OMVs generated? Is OMV biogenesis a spontaneous process or a regulated one? If the process is regulated, what signalling pathways are involved? A comprehensive understanding of their biogenesis may help us tackle their low yield and complex composition. These concerns require fresh approaches. As previously mentioned, OMVs include DNA and RNA [33,34]. None of the current OMV biogenesis models account for nucleic acid packing, which must be transported into the periplasm and over the peptidoglycan layer to be incorporated in an OMV. However, cell lysis releases RNA and DNA that can interfere with the purity of OMVs. Understanding the mechanism by which DNA or RNA reaches the bacterial periplasm could provide valuable insights into the biological functions of OMVs.

A primary issue in the production of OMV-based vaccine platforms is to uphold rigorous quality control and guarantee uniformity and reproducibility for each manufacturing batch [227]. The transition of modified OMVs from laboratory to clinical use would require an in-depth knowledge of potential adverse consequences, their safety profiles and long-term effects in various patient populations. Establishing standardized production protocols and quality control measures is crucial for achieving scalability and consistent reproducibility of these advanced therapies. Future investigations should focus on refining engineering strategies to enhance the specificity and immunogenicity of OMVs, potentially through the identification of neoantigens or the application of cutting-edge genome editing technologies. Furthermore, it is imperative to verify the appropriate bacterial species for manufacturing and to thoroughly assess their activity and attributes (e.g., surface indicators and expressed products) while utilizing bacteria or bacterial components for drug administration. Furthermore, novel separation and purification techniques need to be developed to consistently isolate OMVs from culture medium with improved productivity and purity for large-scale production. Two potential solutions could include a membrane bioreactor with integrated separation features or the use of engineered purification tags on OMVs.

Importantly, our comprehension of the working mechanisms of OMVs in mice remains restricted, and our understanding of their actions in humans is even more constrained. Additional studies are required to gain a deeper understanding of the mechanisms underlying OMV activity, which will contribute to the creation of more efficient OMV-based vaccines. Clinical trials will play a crucial role in confirming preclinical results. While the OMV platform has shown promise in preclinical research for various diseases, only a few OMV vaccines have advanced to clinical trials. This is likely due to the relatively recent development of the OMV platform in comparison to conventional vaccine technologies, regulatory hurdles, and the complexities involved in analyzing OMV-based vaccines compared to well-established subunit vaccines. In essence, collaborative endeavours among clinicians, scientists, and industry stakeholders are crucial to surmount current difficulties, expedite translational initiatives, and advance engineered OMVs into having a broader clinical adoption.

## Figures and Tables

**Table 1:** OMV-based vaccine technology: strengths and limitations compared to other platforms.

Vaccine Platform	Particle Size	Material Properties	Advantages	Disadvantages	Examples	Referenes
Bacterial Ghosts	40–200 nm	Empty envelopes of Gram-negative bacteria	High security; natural immunostimulatory properties	Poor efficiency; complicated production; ambiguous impacts in human application	As of 2025, no human bacterial ghost-based vaccines are fully licenced	[65]
DNA Vaccines	Variable	Plasmid DNA-encoding target antigens	Induce both humoral and cellular immunity; stable; easy to produce	Lower immunogenicity in humans; requires specialized delivery devices or methods	ZyCoV-D (COVID-19 vaccine)	[66]
Inactivated/Killed Vaccines	50–200 nm	Chemically or physically inactivated whole pathogens	Safe; cannot revert to virulence; proven efficacy; easier mass production	Weaker immune response; requires boosters	Polio vaccine, Influenza vaccine	[67]
Liposome-based Vaccines	10 nm-hundreds nm	Phospholipid vesicles	Well-studied; excellent compatibility with biological systems; minimal immune response	Leakage of drug content; inefficient loading capacity; complicated quality control process	Epaxal (Hepatitis A vaccine)	[68]
Live Attenuated Vaccines	~30 nm (small viruses) to 5 µm (bacterial vaccines like BCG)	Weakened (attenuated) version of the pathogen	Elicit strong, long-lasting immunity; stimulate both humoral and cellular responses; often require fewer doses; do not require adjuvants	Risk of reversion to virulence; not suitable for immunocompromised individuals; require cold chain storage	Measles, mumps, and rubella (MMR) vaccine, Varicella (chickenpox) vaccine, BCG vaccine (for tuberculosis)	[69]
mRNA Vaccines	50–150 nm	Lipid nanoparticle-encapsulated mRNA	Highly adaptable; rapid production; strong adaptive immune response	Stability issues; cold chain storage required	Pfizer COVID-19 vaccine (Comirnaty), Moderna COVID-19 vaccine (Spikevax)	[70,71]
Outer-Membrane Vesicles (OMVs)- based Vaccines	30–200 nm	Outer-membrane vesicles of Gram-negative bacteria	Naturally immunogenic; good adjuvant properties; comparatively easy production; cost effective; easily modifiable to express various antigens; low risk of evolutionary escape	Inconsistent antigen presentation efficiency; low yields with some strains	Bexsero (meningococcal disease), VA-MENGOC-BC (meningococcal disease)	[49,63]
Subunit Vaccines (Polysaccharide vaccines, conjugate vaccines, and protein-based vaccines)	Variable	Contain only specific pieces (subunits) of the pathogen, typically proteins or polysaccharides	Good safety profiles; well-characterized; can be combined with adjuvants	Require adjuvants for strong immunity; costly production	Haemophilus influenzae type B (Hib) vaccine, Shingles vaccine	[72]
Toxoid Vaccines	N/A (soluble proteins)	Inactivated bacterial toxins	Good safety profiles; stable; long-lasting immunity	May require boosters; weak innate immune response	Tetanus vaccine, Diphtheria vaccine	[73]
Viral Vector Vaccines	Variable	Modified virus to deliver genetic material encoding an antigen	High transduction efficiency; induces robust immune responses; can target specific cells	Pre-existing immunity to vector; potential safety concerns; complex manufacturing	Ebola vaccine, COVID-19 vaccine (AstraZeneca and Johnson & Johnson)	[74,75]
Virus-Like Particles (VLPs)-based Vaccines	20–200 nm	Non-infectious viral protein structures	High immunogenicity; mimic native viruses; strong B- and T-cell responses	Costly and complex production; requires specialized expression systems	Gardasil/ Cervarix (HPV vaccines), Hecolin (Hepatitis E vaccine)	[73,76]

**Table 2:** Comparative overview of native, detergent-extracted, and genetically modified OMVs.

Feature	Native OMV	Detergent-Extracted OMV	Modified OMV	References
Production Method	Naturally secreted by Gram-negative bacteria	Vesicles extracted from bacterial cells using detergents	Produced through the genetic modification of bacterial strains to enhance vesicle release or introduce foreign antigens; the development of modified strains can be difficult depending upon the type of modification; risk of off-target or secondary mutations during strain development	[114,119]
Scalability	Limited production output depending on the bacterial species and strain used	High yield due to enhanced release through detergent action	High yield facilitated by strain engineering	[49,120]
Ease of Purification	Streamlined and cost-effective purification process	Purification process can be more complex compared to other OMV types	Simple and cost-effective purification	[49,120]
Safety Profile	Contains unaltered pathogen-associated molecular patterns (PAMPs), including LPS, contributing to high reactogenicity	Detergent treatment lowers LPS and lipoprotein levels, reducing endotoxicity	Genetic detoxification of lipid A and deletion of unwanted toxins and antigens enhances safety	[121]
Immunostimulatory Capacity	Exhibits self-adjuvanting effects due to native PAMPs	Reduced immunogenic potential due to lower PAMP content	Preserves adjuvanticity depending on the degree of PAMP modification or removal	
Antigen Presentation	May exhibit low levels of relevant protective antigens	Detergent use can lead to loss of key antigens such as lipoproteins and LPS	Allows for the overexpression of conserved antigens, enabling broader immune coverage	[40,122,123]
Breadth of Immune Protection	Often induces strain-specific immunity with limited cross-protection	Tends to remain strain-specific with minimal species-wide protection	Capable of expressing homologous and heterologous antigens, providing cross-strain and cross-species immunity	[114]

**Table 3:** An overview of various OMV-based vaccines under different stages of development.

Developmental Status	Disease(s)	Pathogen(s)	Vaccine Name	References
Licenced	Diphtheria, tetanus, Whooping cough, Hib infections, and Hepatitis B	Diphtheria, *Clostridium tetani*, *Bordetella pertussis*, Poliomyelitis, *Haemophilus infuenzae* type b, and Hepatitis B	Vaxelis	[127]
	Hib disease and Hepatitis B	*H. infuenzae* type b and Hepatitis B	Procomvax/Comvax * (PRP-OMPC and hepatitis B)	[128]
	Hib disease	*H. infuenzae* type b	PedvaxHib (PRP-OMPC)	[129,130]
	Meningitis	*Neisseria meningitidis* B	Bexsero (4CMenB)	[131,132]
	Meningitis	*N. meningitidis* B	MenZB (NZ dOMV)	[133]
	Meningitis	*N. meningitidis* B	VA-MENGOC-BC	[134,135]
Phase II Clinical Trials	Gonorrhea	*N. gonorrhoea*	*Neisseria gonorrhoeae* GMMA (NgG)	[136] NCT05630859
	Shigellosis	*Shigella sonnei*, *Shigella flexneri* 1b, 2a, and 3a	altSonfex1-2-3	[137,138,139]
Phase I Clinical Trials	COVID-19	SARS-CoV-2	Avacc 10	NCT05604690
	Invasive non-typhoidal Salmonella disease	Invasive non-typhoidal *Salmonella*	iNTS-GMMA	[140,141]
	Meningitis	*N. meningitidis* B	MenPF-1	[142] NCT01640652
	Meningitis	*N. meningitidis* B	Native outer-membrane vesicle (NOMV) vaccine	[143]
Preclinical Studies (Selective Examples)	Acute otitis media, Sinusitis, and Bronchitis	Non-typeable *H. influenzae*	-	[144]
	Anthrax	*Bacillus anthracis*		[145]
	Brucellosis	*Brucella melitensis*	-	[146]
	Chlamydia	*Chlamydia muridarum*	-	[147]
	Cholera	*Vibrio cholerae*	-	[54,148,149,150]
	Chronic periodontitis	*Porphyromonas gingivalis*	-	[151]
	COVID-19	SARS-CoV2	-	[152]
	Diarrhea	Enterotoxigenic *Escherichia coli* (ETEC)	-	[59]
	Gonorrhea	*Neisseria gonorrhoea*	Avacc11	[153]
	Gonorrhea	*N. gonorrhoea*	-	[154,155]
	Influenza	Influenza A virus	-	[156]
	Lung infections	*Klebsiella pneumoniae*	-	[157,158]
	Lyme	*Borrelia burgdorferi*		[159]
	Melioidosis	*Burkholderia pseudomallei*	-	[160]
	Meningitis	*N. meningitidis* B	-	[161]
	Meningitis	*Neisseria lactamica*	-	[162]
	Tuberculosis	*Mycobacterium tuberculosis*	-	[163]
	Pneumonia, Meningitis, and Sepsis	*Acinetobacter baumannii*	-	[164]
	Tularemia	Francisella (different strains)	-	[165]
	Typhoid fever, Gastroenteritis	*Salmonella* Typhimurium	-	[166]
	Whooping cough	*B. pertussis*	-	[164,167,168]

* Licence not renewed for commercial reasons; there are no safety concerns linked to that decision.

**Table 4:** Overview of multi-antigen and multi-pathogen vaccines based on OMVs.

No.	Pathogens	Antigens	OMV Backbone	Developmental Status	Major Study Outcomes	References
1	*Actinobacillus* *Pleuropneumoniae*	A trivalent Apx-fusion protein	*Escherichia coli*	Preclinical	Immunization in mice led to elevated antigen-specific IgG levels and a balanced Th1/Th2 cytokine response, resulting in significant protection against challenges with serotypes 1 and 7	[342]
2	*Chlamydia trachomatis*	Multi-epitope polypeptide of *C. trachomatis* major outer membrane protein (MOMP)	*Salmonella enterica* serovar Typhimurium	Preclinical	Vaccine induced robust systemic and mucosal immune responses in mice, characterized by high levels of specific IgG and IgA antibodies	[163]
3	*Helicobacter pylori*	UreB and CagA of *H. pylori*	*S. enterica* serovar Typhimurium	Preclinical	Vaccination elicited strong humoral and cellular immune responses, including elevated levels of specific antibodies and cytokines, leading to reduced bacterial colonization in the gastric mucosa of mice	[343]
4	HIV-1	HIV-1 Tat and Nef proteins	ClearColi^™^	Preclinical	The engineered OMVs induced potent antigen-specific immune responses in mice, with the significant production of IgG antibodies and activation of CD4^+^ and CD8^+^ T-cells	[344]
5	*Mycobacterium tuberculosis*	ESAT6, Ag85B, and Rv2660c antigens of *M. tuberculosis*	*E. coli* and *S. enterica* serovar Typhimurium	Preclinical	Immunization resulted in strong Th1-type immune responses, characterized by increased IFN-γ production and enhanced protection against *M. tuberculosis* challenge in mice	[163]
6	*Neisseria meningitidis*	NadA, NHBA and fHbp from *N.* *meningitidis* serogroup B	*Neisseria* *cinerea*	Preclinical	Vaccine elicited bactericidal antibodies against multiple *N. meningitidis* serogroup B strains, demonstrating cross-protective potential in murine models	[345]
7	SARS-CoV-2	NG06 fragment and the receptor-binding domain (RBD) of the spike protein	Lumen of *E. coli* Nissle 1917 OMVs	Preclinical	The bivalent OMV vaccine induced strong neutralizing antibody responses and T-cell activation, providing protection against SARS-CoV-2 challenge in mice	[346]
8	Shiga toxin-producing *E. coli* (STEC)	O-antigen polysaccharides of *E. coli* O26, O45, O145, and O103	*S. enterica* serovar Typhimurium	Preclinical	Immunization with the multivalent OMVs elicited robust antibody responses and conferred protection against multiple STEC serotypes in murine models	[347]
9	*Staphylococcus aureus*	Five protective antigens of *S. aureus* as fusions to a lipoprotein leader sequence	*E. coli*	Preclinical	Vaccine induced strong antigen-specific antibody responses and provided significant protection against *S. aureus* infection in mice	[263]
10	*S. aureus*	ClfAY338A, LukE, SpAKKAA, and HlaH35L	*E. coli* BL21(DE3)Δ60	Preclinical	Multivalent OMVs elicited functional antibodies capable of neutralizing key virulence factors, leading to enhanced survival rates in infected mice	[348]
11	*Campylobacter jejuni*, *S. enterica* ser. Typhimurium and *S. enterica* ser. Enteritidis	Isolated OMVs from each pathogen	*C. jejuni*, *S. enterica* ser. Typhimurium and *S. enterica* ser. Enteritidis (OMV pooling)	Preclinical	The trivalent OMV vaccine induced broad-spectrum immune responses and conferred protection against all three pathogens in murine models	[349]
12	Enterotoxigenic *E. coli* (ETEC) and *Shigella flexneri*	ETEC enterotoxin B	*S. flexneri*	Preclinical	Vaccine elicited strong mucosal and systemic immune responses, providing protection against both ETEC and *Shigella* infections in mice	[350]
13	*Enterotoxigenic E. coli* (ETEC) and *Shigella sonnei*	*E. coli* antigens SslE and FdeC	*S. sonnei*	Preclinical	Immunization resulted in robust antibody responses against both pathogens	[114]
14	Enterotoxigenic *E. coli* (ETEC) and *Vibrio cholerae*	Lipopolysaccharide modified and toxin negative OMVs from both pathogens	*V. cholerae* and ETEC (OMV pooling)	Preclinical	The dual-OMV vaccine induced cross-protective immune responses, with significant reductions in bacterial colonization observed in vaccinated mice	[351]
15	Influenza A virus (IAV) and Middle East Respiratory Syndrome (MERS) Virus	H1 hemagglutinin antigen of IAV and receptor binding domain (RBD) of MERS	*E. coli*	Preclinical	The bivalent OMV vaccine elicited strong neutralizing antibody responses against both viruses, providing protection in murine challenge models	[352]
16	*N. meningitidis* and *S. enterica* ser. Typhimurium	MenA and MenC oligosaccharides	*S. enterica* ser. Typhimurium	Preclinical	Vaccine-induced robust antibody responses against both pathogens, demonstrating potential for dual protection in mice	[114]
17	*N. meningitidis* and *Neisseria gonorrhoeae*	fHbp and NHBA-2 of *N. meningitidis* and NHBA-542 of *N. gonorrhoeae*	*Neisseria cinerea*	Preclinical	Immunization elicited cross-reactive antibodies capable of targeting both *N. meningitidis* and *N. gonorrhoeae*	[155]
18	*S. enterica* serovar Paratyphi A and *S. enterica* serovar Typhi	*S.* Paratyphi A homologous O:2 antigens and S. Typhi heterologous Vi	*S. enterica* serovar Paratyphi A	Preclinical	Vaccine induced a strong antibody response against both Vi and O:2, and these antibodies were functional in a serum bactericidal assay	[198]
19	*S. enterica* ser. Typhimurium and Influenza A Virus (IAV)	*S. enterica* ser. Typhimurium OmpA and SseB, and H-stalk protein H5 of IAV	*Bacteroides thetaiotaomicron*	Preclinical	Vaccine induced antigen-specific immune and antibody responses in both mucosal tissues and systemically against both bacterial and viral pathogens	[224]
20	*S. flexneri* 2a, *S. flexneri* 3a, *S. flexneri* 6 and *S. sonnei* I	OMVs from all strains mixed in 1:1:1:1 ratio	*Shigella*	Preclinical	Vaccine induced broad-spectrum immune responses, providing protection against multiple *Shigella* serotypes in murine models	[301]

## Data Availability

No new data were created or analyzed in this study. Data sharing is not applicable to this article.
